# Aspartate β-hydroxylase Regulates Expression of *Ly6* Genes

**DOI:** 10.7150/jca.90422

**Published:** 2024-01-01

**Authors:** Madiha Kanwal, Jana Smahelova, Barbora Ciharova, Shweta Dilip Johari, Jaroslav Nunvar, Mark Olsen, Michal Smahel

**Affiliations:** 1 Department of Genetics and Microbiology, Faculty of Science, Charles University, BIOCEV, Vestec, Czech Republic.; 2 Department of Pharmaceutical Sciences, College of Pharmacy - Glendale, Midwestern University, Glendale, AZ, USA.

**Keywords:** Tumorigenesis, Ly6 family, ASPH inhibitor, RNA sequencing

## Abstract

**Background:** Overexpression of aspartate β-hydroxylase (ASPH) in human tumors contributes to their progression by stimulating cell proliferation, migration, and invasion. Several signaling pathways affected by ASPH have been identified, but the high number of potential targets of ASPH hydroxylation suggests that additional mechanisms may be involved. This study was performed to reveal new targets of ASPH signaling.

**Methods:** The effect of ASPH on the oncogenicity of three mouse tumor cell lines was tested using proliferation assays, transwell assays, and spheroid invasion assays after inhibition of ASPH with the small molecule inhibitor MO-I-1151. ASPH was also deactivated with the CRISPR/Cas9 system. A transcriptomic analysis was then performed with bulk RNA sequencing and differential gene expression was evaluated. Expression data were verified by quantitative PCR and immunoblotting.

**Results:** Inhibition or abrogation of ASPH reduced proliferation of the cell lines and their migration and invasiveness. Among the genes with differential expression in more than one cell line, two members of the lymphocyte antigen 6 (*Ly6*) family, *Ly6a* and *Ly6c1*, were found. Their downregulation was confirmed at the protein level by immunoblotting, which also showed their reduction after ASPH inhibition in other mouse cell lines. Reduced production of the Ly6D and Ly6K proteins was shown after ASPH inhibition in human tumor cell lines.

**Conclusions:** Since increased expression of *Ly6* genes is associated with the development and progression of both mouse and human tumors, these results suggest a novel mechanism of ASPH oncogenicity and support the utility of ASPH as a target for cancer therapy.

## Introduction

Cancer is a complex disease induced by genetic and epigenetic alterations leading to aberrant activation of specific signaling pathways critical for cell cycle control, DNA repair, and apoptosis and resulting in uncontrolled cell proliferation, tumor development, and metastasis 1. In this regard, strategically targeted cancer therapies are emerging as treatments aiming to control signaling pathways that regulate cell survival and death 2. Although conventional chemotherapy and radiotherapy remain the treatment of choice for many malignancies, their toxicity and adverse side effects, as well as the development of resistance, support the need to find new targeted therapies 3. Recently, inhibitors that modulate the enzymatic activity of aspartate β-hydroxylase (ASPH) have been shown to be effective therapeutic agents 4-8.

With the exception of extravillous trophoblasts in the placenta 9, ASPH has negligible or very low expression in most normal adult human tissues, but during oncogenesis, it is markedly upregulated by growth factor signaling pathways RAS/RAF/MAPK/ERK, PI3K/AKT, and WNT/β-catenin 4,10,11 and can contribute to the development and progression of malignancies. When upregulated in tumor cells, ASPH was found on the cell surface where its catalytic site located in the C-terminal region is exposed to the extracellular environment and can be recognized and attacked by the host antibody response and monoclonal antibodies 12-14. The presence of antigenic epitopes can also efficiently stimulate T-cell responses specific to tumor cells harboring ASPH 15. ASPH is a transmembrane protein that belongs to the α-ketoglutarate-dependent dioxygenase family 16 and regulates cell functions by post-translational hydroxylation of aspartyl and asparaginyl residues in epidermal growth factor-like protein domains 15-17. ASPH is upregulated in several malignant neoplasms where it propagates a malignant phenotype associated with increased cell proliferation, invasiveness, and metastasis, and with poor clinical prognosis 9,10,16,18.

Notch receptors and their ligands can be hydroxylated with ASPH 10,16 which stimulates Notch signaling and upregulates the Notch-responsive genes such as hes family bHLH transcription factor 1 (HES1), HES with YRPW motif 1 (HEY1), c-myelocytomatosis oncogene (c-Myc), matrix metallopeptidases (MMPs), cyclin D3, and proliferating cell nuclear antigen (PCNA), resulting in enhanced cell proliferation, migration, and invasion 4,19. Furthermore, the proto-oncogene SRC tyrosine kinase (SRC) can be activated by ASPH through direct interaction of ASPH with a disintegrin and metallopeptidase domain 12/15 (ADAM12/15). The activated SRC pathway promotes tumor growth and invasion by stimulation of angiogenesis, invadopodia formation, and extracellular matrix degradation 8.

Notch and SRC signaling are probably major pathways affected by ASPH activation, but there may be other functions of overexpressed ASPH, that can support a malignant phenotype. For instance, dysregulation of glycogen synthase kinase-3β (GSK-3β) has been linked to a variety of human diseases, including cancer. Inhibition of ASPH expression or enzymatic activity can influence GSK-3β activity by increasing its phosphorylation, suggesting that ASPH may impact the interaction of GSK-3β with its upstream kinases 6,20. Furthermore, epithelial-mesenchymal transition (EMT) plays a key role in tumor progression and metastasis and recent studies reported that ASPH functioned as a stimulator of EMT through interacting with vimentin, extracellular signal-regulated kinase (ERK), and phosphatidylinositol-3-kinase/protein kinase B (PI3K/AKT) signaling pathways 21,22.

The demonstration that ASPH enzymatic activity promotes tumor growth and metastasis raised the possibility that inhibition or reduction of this activity may be used in cancer therapy. In this regard, several candidate small molecule inhibitors (SMIs) were synthesized as potential inhibitors of ASPH β-hydroxylase activity on the basis of the crystal structure of the catalytic site in the C-terminus of ASPH 23,24. These SMIs showed promising results in *in vitro* and *in vivo* experiments 5,8,25, with up to 80% reduction of ASPH enzymatic activity and a substantial decrease of proliferation, migration, and invasion of tumor cells in different cancer models 4,5.

Despite the advances in understanding the molecular mechanisms involving ASPH, the large number of other potential ASPH hydroxylation targets 14 suggests a possible contribution of additional signaling pathways to ASPH-mediated migration, invasion and metastasis. In the present study, we showed the oncogenic characteristics of ASPH in mouse tumor cell lines and then utilized next-generation RNA sequencing (RNA-seq) to identify candidate ASPH-regulated genes with differential expression after treating these cell lines with the second-generation ASPH inhibitor MO-I-1151. Importantly, the study identified a potential role of ASPH in regulating expression of genes of the Lymphocyte antigen 6 (Ly6) family (*Ly6a* and *Ly6c1*). However, further study is needed to reveal a mechanism of this signaling.

## Materials and methods

### Cell lines

TC-1 cell line (RRID: CVCL_4699) was derived from mouse primary lung epithelial cells transformed with the E6 and E7 oncoproteins of human papillomavirus type 16 (HPV-16) 26; the TC-1/A9 clone (RRID: CVCL_ZW99), a derivative of TC-1 cell line, was selected based on suppressed major histocompatibility complex class I (MHC-I) surface expression 27. MK16/KLL is a highly metastatic cell line derived after transduction of mouse secondary kidney cells with the HPV-16 *E6/E7* oncogenes and activated *H-ras* 28. RMA cells (RRID: CVCL_J385) are lymphoma T cells 29, B16-F10 cells (RRID: CVCL_0159) were derived from melanoma 30, 4T1 cell line (RRID: CVCL_0125) is a model of breast cancer 31, and JUN-3 cell line was derived from fibrosarcoma 32. Human cell lines HeLa (RRID: CVCL_0030), CaSki (RRID: CVCL_1100), and SiHa (RRID: CVCL_0032) were derived from cervical carcinoma, Detroit 562 (RRID: CVCL_1171) from pharyngeal carcinoma and MCF-7 (RRID: CVCL_0031) from breast cancer. RMA, HeLa, SiHa, and CaSki cells were cultured in Roswell Park Memorial Institute 1640 medium (RPMI-1640; Sigma-Aldrich, St. Louis, MO, USA); Detroit 562 cells were cultured in Eagle's minimum essential medium (EMEM; Sigma-Aldrich); and all other cell lines were maintained in Dulbecco's modified Eagle's medium (DMEM; Sigma-Aldrich). The media were supplemented with 10% fetal bovine serum (FBS; Sigma Aldrich), 100 IU/mL penicillin, and 100 μg/mL streptomycin (Biosera, Kansas, MO, USA). Cells were incubated in a humidified incubator at 37°C in an atmosphere containing 5% CO_2_ and harvested at 80% confluency with 0.05% trypsin-EDTA in phosphate-buffered saline (PBS) for further investigations.

To generate TC-1/dASPH cell line with the deactivated *ASPH* gene, ASPH Double Nickase Plasmid (m) (sc-425778-NIC; Santa Cruz Biotechnology, Dallas, TX, USA) was used. TC-1 cells were transfected with plasmid DNA using Lipofectamine 2000 (ThermoFisher Scientific, Waltham, MA, USA) and 48 h after transfection, GFP positive cells were sorted into a 96-well plate by the BD FACS Aria Fusion cell sorter (BD Biosciences, Franklin Lakes, NJ, USA) to obtain individual clones. All the grown clones were subjected to western blot to confirm ASPH knockout.

### MTT assay

To examine the dose-dependent effect of ASPH inhibitors on proliferation of mouse tumor cell lines, the MO-I-1151 inhibitor was employed in the 3-[4,5-dimethylthiazol-2-yl]-2,5-diphenyl-tetrazolium bromide (MTT) assay. The MO-I-1151 inhibitor is a second-generation SMI with improved solubility and inhibitory effect against ASPH enzymatic activity 4,6. TC-1, TC-1/dASPH, TC-1/A9, and MK16/KLL cells were seeded in density of 1×10^4^ cells per well in a 96-well culture plate. Cell lines were treated with the MO-I-1151 inhibitor at concentrations ranging from 0.1 to 20 μM. After incubation for 48 h, MTT (5 mg/mL) was added to the growth media for 4 h, the purple crystals were dissolved in dimethyl sulfoxide (DMSO) for 3 h, and absorbance was measured at wavelength 562 nm by the Infinite M200 Pro microplate reader (TECAN, Mannedorf, Switzerland). The results were expressed as a percentage of absorbance relative to the respective untreated control.

### Wound healing assay

To monitor cell migration, an *in vitro* wound healing assay was performed. Cell lines were seeded in a 6-well culture plates and treated with DMSO or the MO-I-1151 inhibitor (20 μM) for 24 h at 37°C. When the cells formed a monolayer, a scratch was made in the central area of a well by a sterile 200-μl pipette tip, followed by PBS wash to remove cell debris and cultivation in a fresh medium containing DMSO or the MO-I-1151 inhibitor. Cell migration toward the gap area was documented by a Nikon Eclipse TE2000-S microscope (Nikon, Tokyo, Japan) using Hoffman modulation contrast (4× objective). The size of the gap at a selected position was photographed at 0, 6 and 12 h and analyzed by the ImageJ software (www.imagej.org). The ratio of the gap size at 6 and 12 h to the gap size at 0 h was calculated and triplicates were averaged.

### Transwell migration and invasion assay

Uncoated and matrigel precoated transwell chambers (8 μm pore size; Corning, Lowell, MA, USA) in 24-well plates were used to assess cell migration and invasion, respectively. Briefly, 5×10^4^ TC-1, TC-1/dASPH, TC-1/A9, and MK16/KLL cells were suspended in serum-free DMEM, seeded in an upper chamber, and treated with DMSO or the MO-I-1151 inhibitor (20 μM). DMEM supplemented with 10% FBS was added to the lower chamber as a chemoattractant and cells were allowed to migrate for 24 h at 37°C. Then, cells remaining on the upper surface of the filter were removed by a cotton swab and cells that migrated on the lower surface were fixed with 95% ethanol for 15 min and stained with 0.05% crystal violet for 5 min. For quantification of migratory and invasive ability of the cells, 4 fields of the membrane were randomly photographed by an Olympus IX71 microscope (Olympus, Tokyo, Japan) using Hoffman modulation contrast (10× objective). Images were analyzed by the ImageJ software and cell density was measured as pixel intensity, by applying background subtraction and contrast enhancement. Relative pixel intensity was calculated by dividing the mean pixel intensity of the image of cells treated with the MO-I-1151 inhibitor by that of DMSO-treated sample.

### Three-dimensional (3D) spheroid invasion assay

To obtain spheroids, cells were grown in gelled agarose microwells using a 3D Petri Dish (Sigma Aldrich) in DMEM supplemented with 10% FBS and penicillin and streptomycin for 48-72 h. Buffered solution composed of DMEM, 0.375% (w/v) NaHCO_3_, 8.5 mM NaOH, and 15 mM HEPES was mixed in the 3:1 ratio with 1 mg/mL of collagen solution (Cultrex 3-D Culture Matrix Rat Collagen I; R&D Systems, Minneapolis, MN, USA) and spheroids were embedded into 3D collagen matrix. The polymerized collagen matrix was overlaid with culture media supplied with DMSO or the MO-I-1151 inhibitor. Images of the embedded spheroid were taken at 0 and 72 h. The area of control and treated spheroids before and after invasion were calculated by the ImageJ software and at least three spheroids per condition were analyzed in each experiment.

### Colony formation assay

TC-1, TC-1/dASPH, TC-1/A9, and MK16/KLL cells were seeded at a density of 500 cells/well into 6-well plates. Cells were treated with DMSO or the MO-I-1151 inhibitor (20 μM) and incubated at 37⁰C for 7 days. Colonies were fixed with 4% formaldehyde for 15 min and stained with 0.05% crystal violet dye for 5 min. Plates were scanned using the Brother MFC-J5320DW scanner (Brother, Nagoya, Japan) and the colonies were assessed by the ImageJ software.

### Bulk RNA sequencing

Total RNA was isolated with the NucleoSpin RNA kit (Macherey Nagel, Düren, Germany) from TC-1, TC-1/dASPH, TC-1/A9, and MK16/KLL cell lines treated with DMSO or the MO-I-1151 inhibitor (20 μM) for 24 h. The isolation was performed three times following the manufacturer's protocol. Libraries for next-generation sequencing were prepared using the QuantSeq 3´mRNA-Seq Library Prep Kit (FWD) for Illumina (Lexogen, Vienna, Austria) and sequenced on the NextSeq 500 System (Illumina, San Diego, CA, USA) with 75 bp reads at the Core Facility for Genomics and Bioinformatics (Institute of Molecular Genetics, Prague, Czech Republic). Differential gene expression was analyzed using Geneious Prime 2020 (https://www.geneious.com) as described previously 33. Overlap of differentially expressed genes among cell lines was found and visualized with the Venny 2.1 tool (https://bioinfogp.cnb.csic.es/tools/venny/index.html) and enrichment analysis was performed with GSEA 4.2.3 34,35 and gene sets obtained from the Molecular Signature Database 7.2 36,37.

### Quantification of mRNA Expression by RT-qPCR

Total RNA (500 ng) was treated with Dnase I (Jena Bioscience, Germany) and reverse transcribed in a 20-μL reaction using M-MLV Reverse Transcriptase (Promega, Madison, WI, USA) and Random Hexamers (IDT, Leuven, Belgium) according to the manufacturer's instructions. Predesigned KiCqStart® SYBR® Green Primers (Merck, Haverhill, United Kingdom) were used for the detection of *Ly6a, Ly6c1,* Dickkopf WNT signaling pathway inhibitor 2 (*Dkk2*)*,* growth factor receptor bound protein 14 (*Grb14*)*,* heme oxygenase 1 (*Hmox1*)*,* and solute carrier family 14 member 1 (*Slc14a1*) genes. Actin beta (*Actb*); forward primer 5'-CAACTGGGACGACATGGAGAAGAT-3', reverse primer 5'-CATGGCTGGGGTGTTGAAGGTC-3' served as a reference gene for normalization. Amplifications of cDNA of the target genes and the reference gene were performed simultaneously in duplicates in a 384-well microplate format of the CFX384 Touch Real-Time PCR Detection System (Bio-Rad, Hercules, CA, USA) with SYBR green chemistry by the Gene Core - Quantitative and Digital PCR (BIOCEV, Vestec, Czech Republic). Ten microliters of the reaction solution contained 1× Xceed qPCR SG Lo-ROX master mix (IAB, Prague, Czech Republic), 400 nM of each primer and 2 μL cDNA (diluted 2.5×). The following program was used for amplification: 95°C for 3 min followed by 45 cycles of 95°C for 10 s, 60°C for 30 s, and melting curve. Data processing and relative quantification of mRNA expression were performed using the GenEx v6 software (TATAA Biocenter, Goteborg, Sweden).

### Immunoblotting

After 24-h treatment with DMSO or the MO-I-1151 inhibitor, cells were lysed in lysis buffer (62.5 mM Tris pH 6.8, 20% glycerol, 4% (w/v) sodium dodecyl-sulfate (SDS) and protein concentration was quantified by a BCA protein assay (ThermoFisher Scientific). Equal amounts of proteins were resolved by 10% SDS polyacrylamide gel electrophoresis (SDS-PAGE), electroblotted to a polyvinylidene difluoride (PVDF) membrane, and blocked in 5% skimmed milk, followed by incubation with primary antibodies targeting mouse ASPH (cat. no. NBP1-69230, 1:1000; Novus Biologicals, Littleton, CO, USA; PB9478, 1:1000; Boster Biological Technology, Pleasanton, CA, USA); human ASPH (cat. no. NBP1-69229, 1:1000; Novus Biologicals); Notch1 (3608, 1:1000), activated-Notch1 (4147, 1:1000), HES1 (11988, 1:1000), Snail (3879, 1:500), Slug (9585, 1:500),Vimentin (5741, 1:1000), c-Myc (18583, 1:1000), Phospho-c-Myc-Thr58 (46650), SRC (2109, 1:1000), and pSRC-Tyr416 (6943, 1:1000) from Cell Signaling Technology (Danvers, MA, USA); Ly6a/Sca-1 (ab109211, 1:500; Abcam, Cambridge, UK); Ly6c (70362, 1:500; Elabscience, Houston, TX, USA); Ly6D (A17570, 1:1000; ABclonal Technology, Wuhan, China); Ly6K (sc-393560, 1:500; Santa Cruz Biotechnology; Dallas, TX, USA) and GAPDH (MA5-157380, 1:2000; ThermoFisher Scientific). After washing with PBS + 0.05% Tween, the membranes were incubated with horseradish peroxidase (HRP)-linked secondary antibody for 2 h at room temperature and washed. Protein bands were visualized by staining with the Immobilon ECL ultra-western HRP substrate (Millipore, Billerica, MA, USA) and detected by the Amersham Imager 600 (GE Healthcare, Chicago, IL, USA).

### Statistical analyses

All results were presented as the mean ± the standard error of the mean (SEM). Statistical differences between two groups were determined by the student's *t*-test. Statistical analyses were performed with the Prism software, version 8 (GraphPad Software, San Diego, CA, USA). *P*<0.05 was considered statistically significant.

## Results

### ASPH inhibition impairs proliferation of mouse tumor cell lines

**Figure 1:**
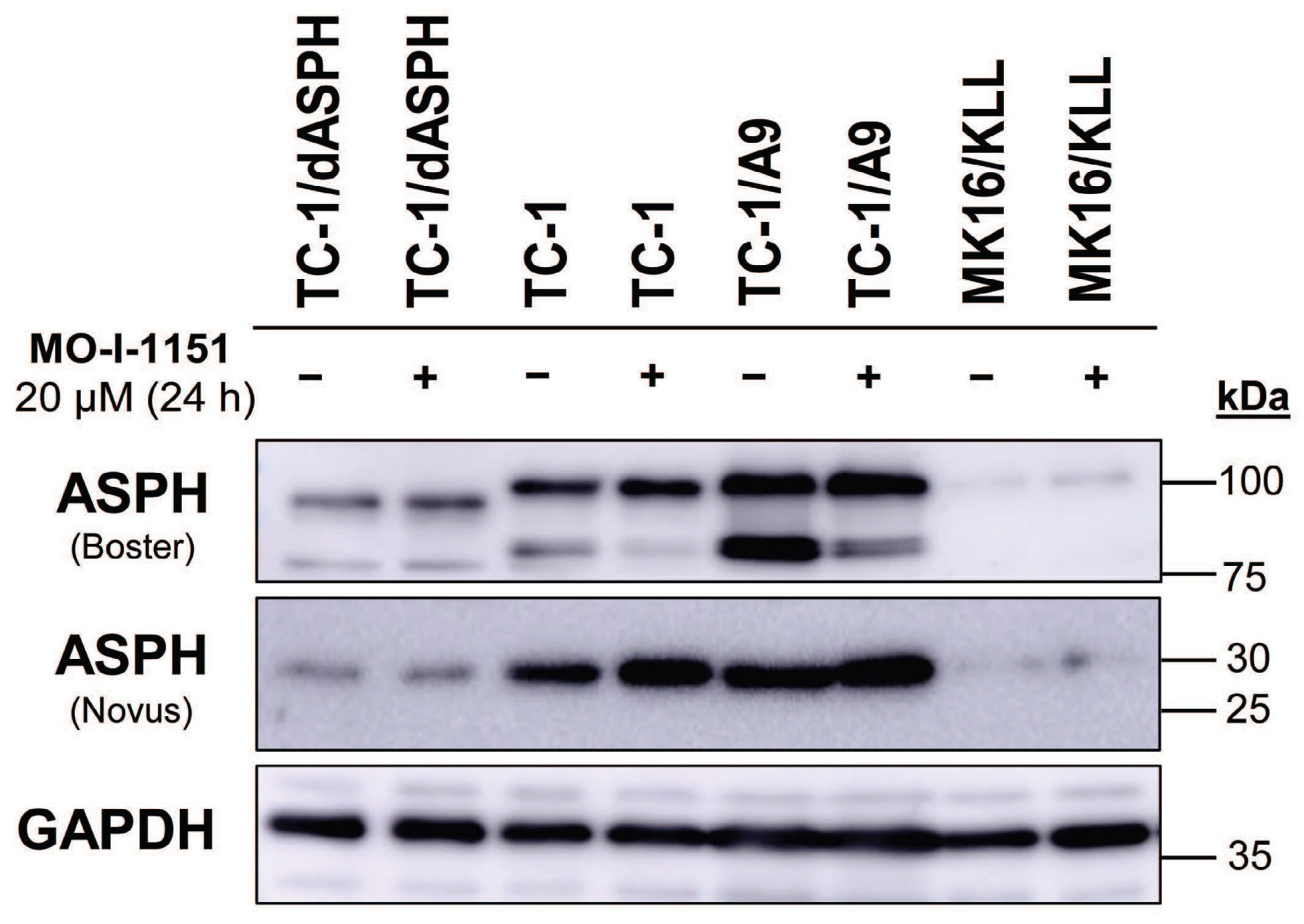
Immunoblotting detection of the ASPH protein. TC-1, TC-1/dASPH, TC-1/A9 and MK16/KLL cells were treated with 20 μM MO-I-1151 inhibitor for 24 h and DMSO was used as a control. Glyceraldehyde-3-phosphate dehydrogenase (GAPDH) was used as an internal control. Antibodies applied for ASPH staining are indicated.

To investigate the function of ASPH in mouse tumor cell lines, we knocked out the *ASPH* gene in TC-1 cells by using the CRISPR/Cas9 system. Then, we examined ASPH production in the selected clone TC-1/dASPH by immunoblotting (Figure 1). While the Novus antibody, that recognizes an epitope at the N-terminus of ASPH, showed markedly reduced production of the 30 kDa (junctin) isoform, the Boster antibody, that binds to the C-terminus of ASPH, detected 2 bands (about 100 and 85 kDa) that were also reduced and were shifted to lower molecular weights. We suppose that the upper band represents full-length ASPH and the lower band results from an alternative splicing. These changes in ASPH expression were associated with markedly decreased cell proliferation (52 h doubling time *vs* 18 h for the parental TC-1 cells) which was further impaired after treatment with the MO-I-1151 inhibitor. When compared with TC-1 cells, the levels of the detected ASPH isoforms were substantially lower in MK16/KLL cells. In TC-1/A9 cells, the levels of the isoforms stained with the Novus antibodies were comparable to those in TC-1 cells, but the isoforms detected with the Boster antibody were increased in TC-1/A9 cells, particularly the lower-weight isoform that was the only isoform reduced in TC-1 and TC-1/A9 cells after treatment with the MO-I-1151 inhibitor.

**Figure 2:**
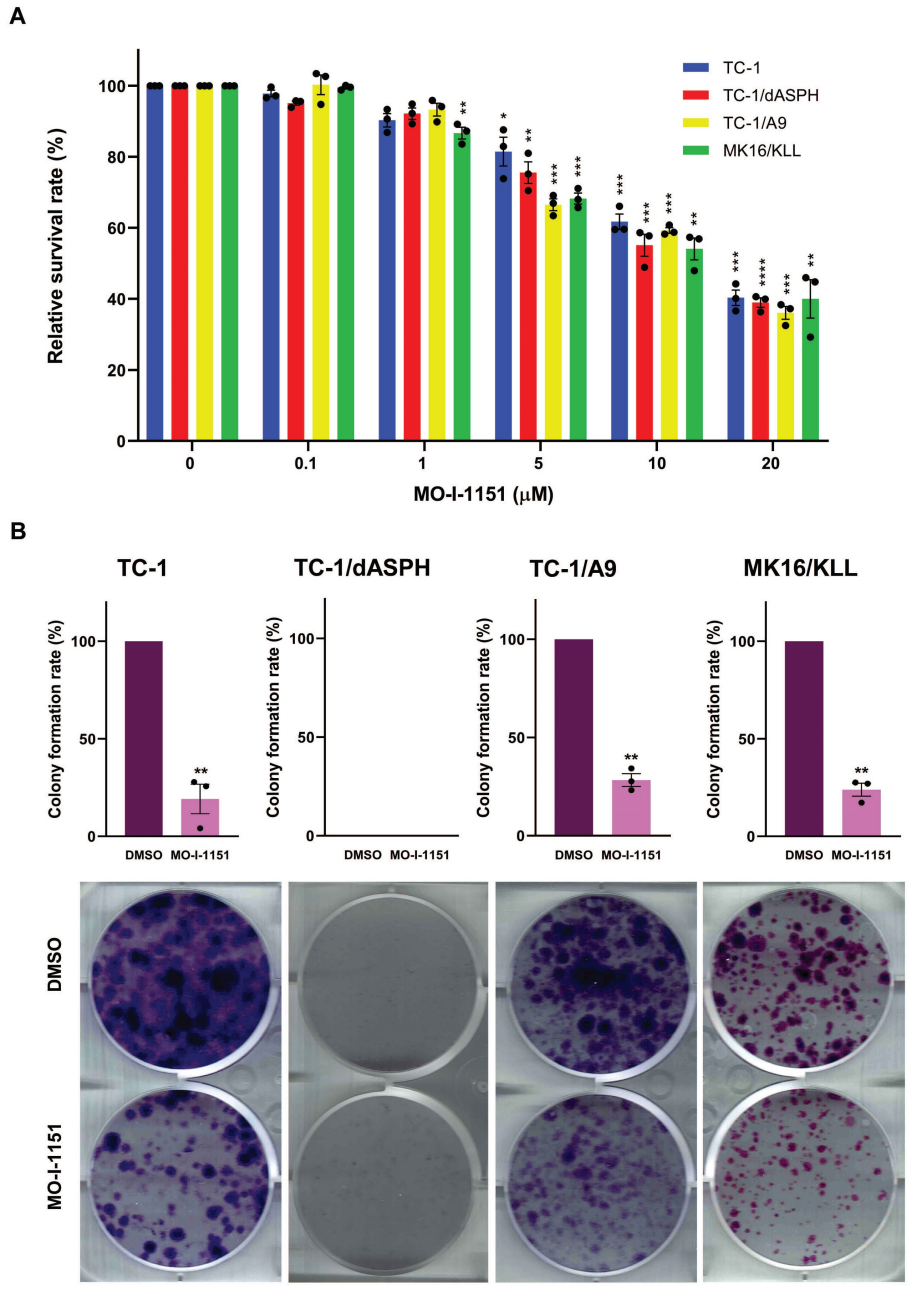
The effect of ASPH inhibition on cell proliferation.** (A)** TC-1, TC-1/dASPH, TC-1/A9 and MK16/KLL cells were treated with the MO-I-1151 inhibitor at concentration 0.1, 1, 5, 10 and 20 μM for 48 h and then subjected to an MTT assay or **(B)** incubated with MO-I-1151 at 20 μM for 7 days, stained with crystal violet and photographed. Images were quantified by ImageJ software. DMSO was used as a control. Data represents the mean ± SEM of three independent experiments. Statistical significance refers to the comparison with the non-treated (DMSO) samples. * *p*<0.05, ** *p*<0.01, *** *p*<0.001, **** *p*<0.0001 by *t*-test.

We then examined the anti-proliferative ability of the MO-I-1151 inhibitor in the studied mouse tumor cell lines by the MTT assay. All cell lines treated with increasing concentrations of the MO-I-1151 inhibitor showed significantly reduced proliferation in a dose dependent manner (Figure 2A). The concentrations of MO-I-1151 which inhibited 50% of the growth (IC_50_) were 15.8, 14.6, 14.0 and 14.3 μM for TC-1, TC-1/dASPH, TC-1/A9, and MK16/KLL cells, respectively.

The colony formation assay was also performed to further assess the impact of inhibition of ASPH hydroxylase activity on the cell proliferation. This assay showed the inhibitory effect of MO-I-1151 on clonogenic potential of TC-1, TC-1/A9 and MK16/KLL cell lines as the MO-I-1151 treatment reduced the number and size of colonies compared to the control sample (Figure 2B). Both MO-I-1151 treated and untreated TC-1/dASPH cells failed to form any colonies. These results suggest that ASPH contributes to the stimulation of proliferation in TC-1, TC-1/A9, and MK16/KLL cells.

### ASPH inhibition suppresses migration and invasion of mouse tumor cell lines

**Figure 3:**
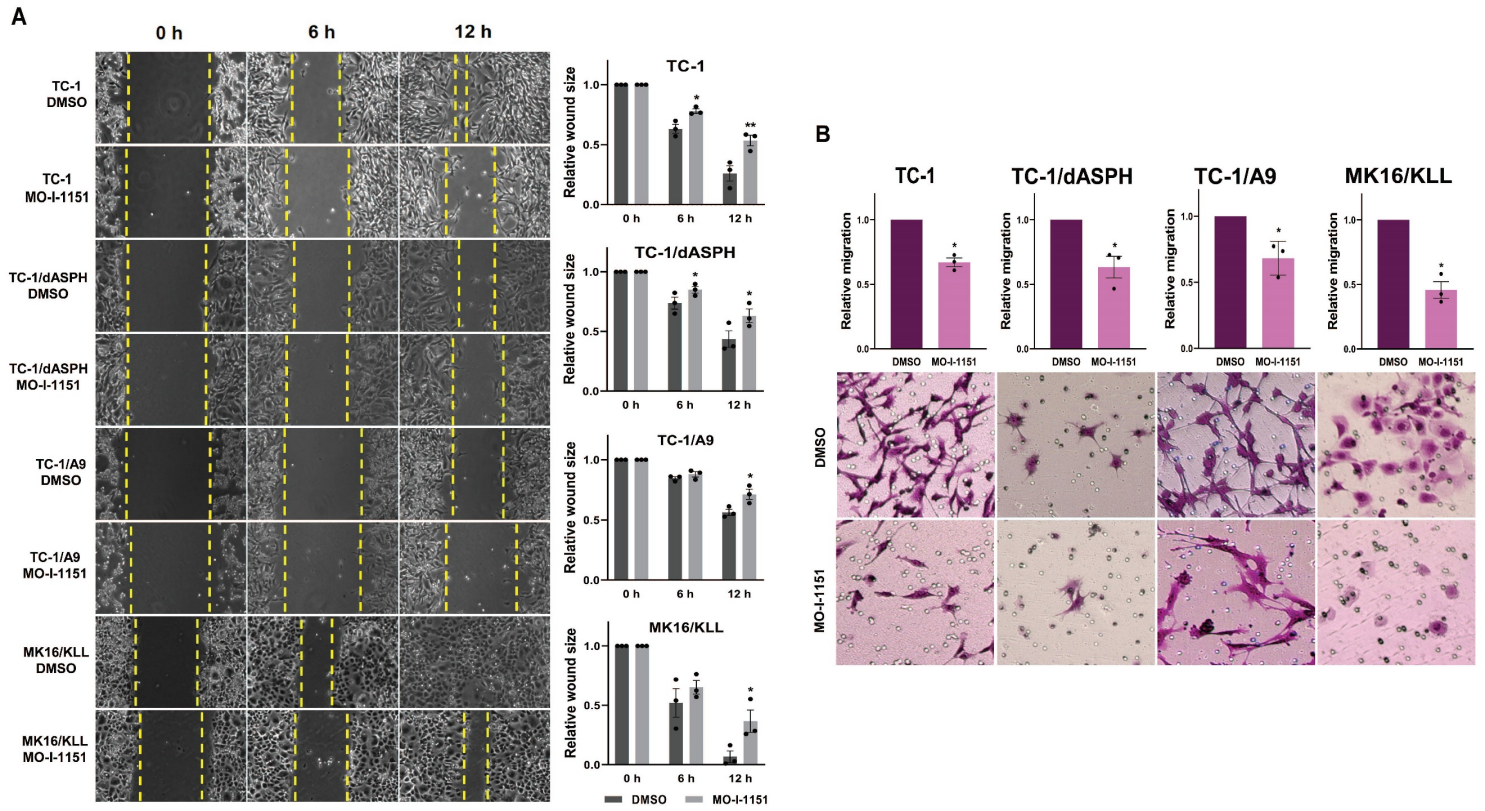
The effect of ASPH inhibition on cell migration.** (A)** Confluent TC-1, TC-1/dASPH, TC-1/A9 and MK16/KLL cells were incubated with 20 μM MO-I-1151 for the indicated times. The migration was measured by the area of the wound made in cells and images were taken at 0, 6 and 12 h and images were quantified using ImageJ software. **(B)** Cell lines were treated with 20 μM MO-I-1151 in transwell chambers. After 24 h, the cells were fixed and stained. Microscopic images were quantified by ImageJ software. DMSO was used as a control. Data represents the mean ± SEM of three independent experiments. Statistical significance refers to the comparison with the non-treated (DMSO) samples. * *p*<0.05*,* ** *p*<0.01 by *t*-test.

We first performed an *in vitro* wound healing assay to assess the ability of our mouse tumor cells to migrate under the influence of the ASPH inhibitor. MO-I-1151 treatment of TC-1, TC-1/dASPH, TC-1/A9 and MK16/KLL cells significantly reduced migration of all cell lines into the denuded area compared to their respective DMSO controls (Figure 3A). To further investigate the role of the MO-I-1151 inhibitor in the impairment of cell migration, we tested the cells in a transwell migration assay and found that the migration of all cell lines was also significantly inhibited after MO-I-1151 treatment (Figure 3B). In addition, migration of TC-1 cells was markedly reduced in both assays after *ASPH* deactivation in TC-1/dASPH cells.

**Figure 4:**
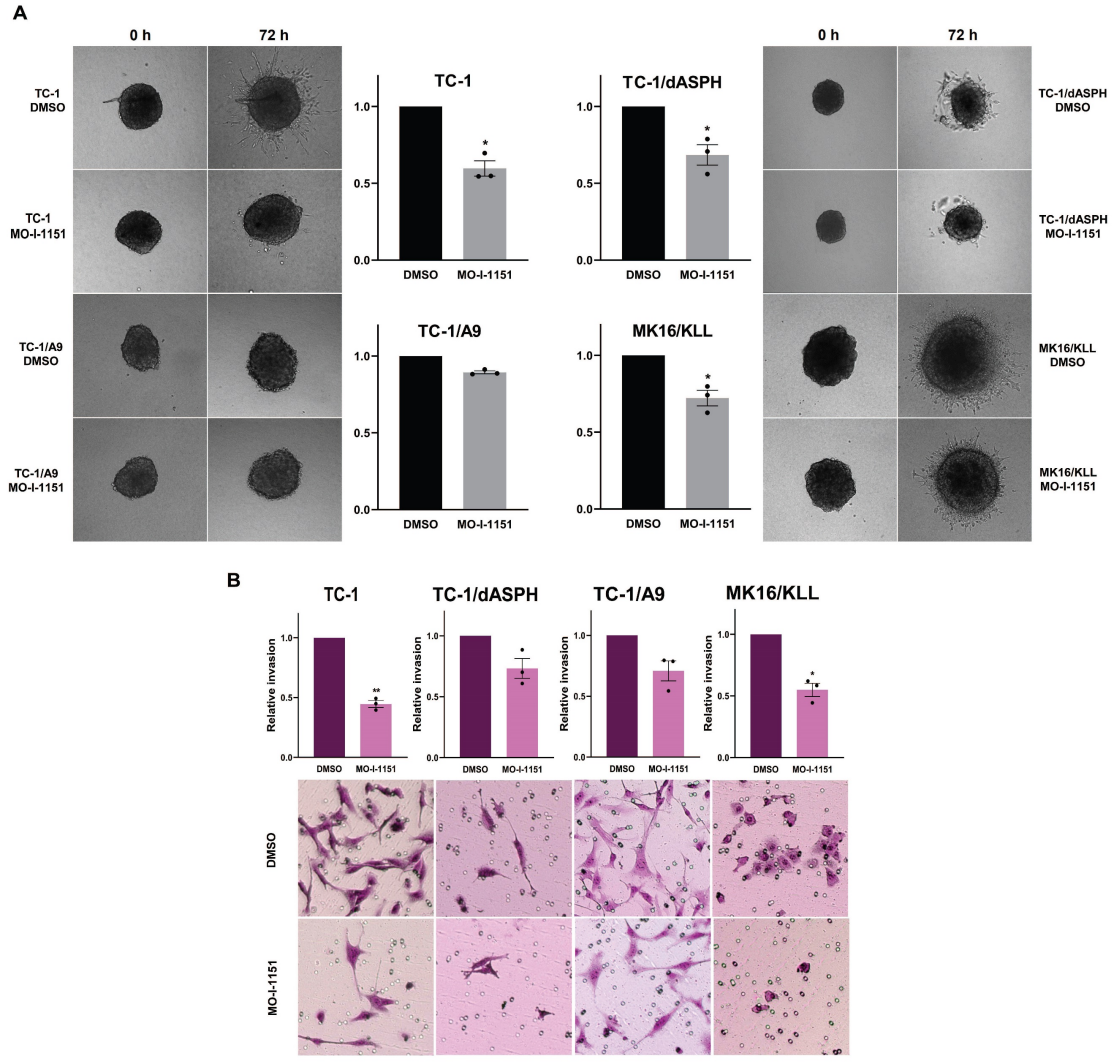
The effect of ASPH inhibition on cell invasions. **(A)** Spheroids were embedded into a 3D collagen matrix and treated with 20 μM MO-I-1151. Images were taken at 0 and 72 h and quantified by ImageJ software. **(B)** Cell lines were treated with 20 μM MO-I-1151 in pre-coated Matrigel transwell chambers. After 24 h, the cells were fixed and stained. Microscopic images were quantified by ImageJ software. DMSO was used as a control. Data represents the mean ± SEM of three independent experiments. Statistical significance refers to the comparison with the non-treated (DMSO) samples. * *p*<0.05, ** *p*<0.01 by *t*-test.

Next, to investigate whether targeting ASPH hydroxylase activity with the MO-I-1151 inhibitor may impede invasion of the tumor cell lines studied, we used a 3D spheroid model. The MO-I-1151 inhibitor significantly reduced the invasion of TC-1, TC-1/dASPH, and MK16/KLL cell lines. TC-1/A9 cells did not show an invasion potential in this assay (Figure 4A).

To further confirm the effect of the MO-I-1151 inhibitor on invasion activity of the tumor cell lines, we also tested the cells in a transwell invasion assay. The numbers of cells invading through a transwell membrane were significantly reduced for TC-1 and MK16/KLL cells after MO-I-1151 treatment compared to their respective DMSO controls. The inhibition of ASPH hydroxylase activity in TC-1/dASPH and TC-1/A9 cells resulted in reduced invasion in repeated experiments but this decrease was not significant (Figure 4B). In addition, TC-1/dASPH cells exhibited significantly (p=0.046) lower invasion in comparison with TC-1 cells. Taken together, these results suggest an ASPH role in migration and invasiveness of TC-1, TC-1/A9, and MK16/KLL cells.

### Ambiguous effect of ASPH inhibition on signaling pathways

**Figure 5:**
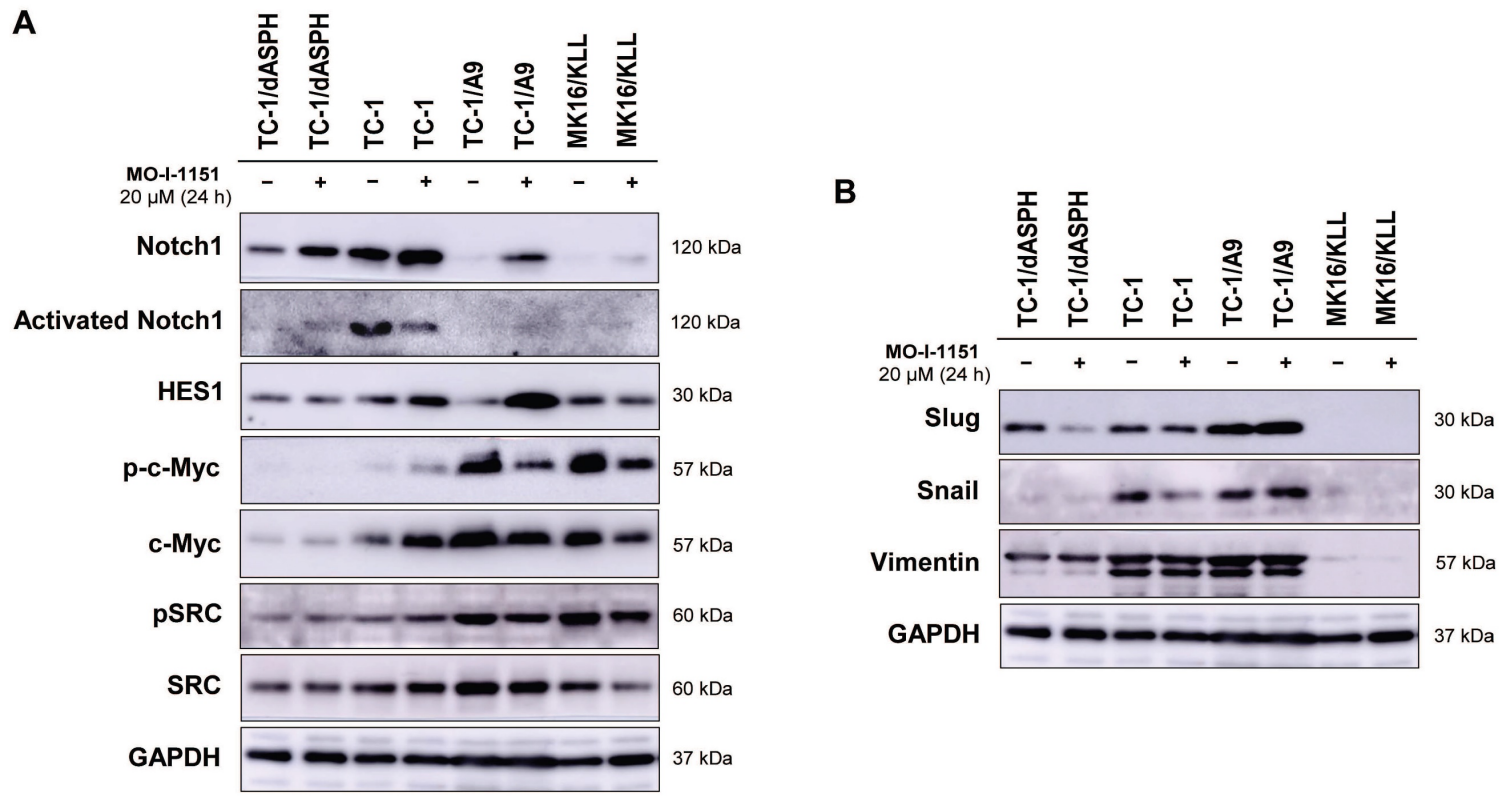
The effect of ASPH inhibition on cellular signaling. Cells were treated with 20 μM MO-I-1151 for 24 h (DMSO was used as a control), and protein samples were collected and subjected to SDS-PAGE electrophoresis and immunoblotting. Glyceraldehyde-3-phosphate dehydrogenase (GAPDH) was used as an internal control. (A) Notch and SRC pathways, (B) epithelial-mesenchymal pathway.

Since ASPH has been shown to modulate the Notch and SRC pathways, we investigated whether modification of cell proliferation, migration, and invasion induced in the tested mouse tumor cell lines by knockout of *ASPH* and/or inhibition of the ASPH hydroxylase activity with the MO-I-1151 inhibitor can be associated with alterations in these pathways. Interestingly, the cells treated with the SRC inhibitor dasatinib downregulated SRC signaling (Figure S1), but the MO-I-1151 inhibitor did not decrease the phosphorylation status of SRC in TC-1/dASPH and TC-1 cells and only slightly reduced the phosphorylated SRC in TC-1/A9 and MK16/KLL cell lines, but this reduction was accompanied by downregulation of total SRC (Figure 4A). Next, the detection of Notch1 expression showed that the level of total Notch1 was very different in the examined tumor cell lines and this level was increased in all cell lines after treatment with the MO-I-1151 inhibitor. The activated Notch1 was only observed in TC-1 cells with the highest level of total Notch1 and was reduced by the treatment with the MO-I-1151 inhibitor. However, the Notch1 downstream target HES1 was increased in TC-1 cells after the treatment with MO-I-1151 (Figure 5A). Furthermore, the TC-1/A9 cells with a low level of Notch1 exhibited a high level of HES1 following treatment with the MO-I-1151 inhibitor, suggesting Notch1-independent stimulation of HES1 expression. The c-Myc is another important transcriptional target of Notch1 signaling 38. Compared to the respective controls, the treatment with the MO-I-1151 inhibitor induced a decrease of both phosphorylated and total c-Myc in TC-1/A9 and MK16/KLL cells, but an opposite effect was observed in TC-1 cells where c-Myc and p-c-Myc levels were increased in MO-I-1151-treated cells.

As the hydroxylase activity of ASPH is known to upregulate the EMT pathway 22, we also examined the expression of EMT associated markers. The ASPH knockout in TC-1/dASPH cell line led to the downregulation of mesenchymal markers Snail and vimentin as compared to parental TC-1 cells that only reduced Snail production after treatment with the MO-I-1151 inhibitor. Another mesenchymal marker Slug only showed downregulation in TC-1/dASPH cells after the treatment with the MO-I-1151 inhibitor (Figure 5B). In TC-1/A9 cells, the MO-I-1151 inhibitor did not affect any of the detected EMT-related proteins. Moreover, epitheloid MK16/KLL cells did not express any mesenchymal marker investigated in the study. Our findings showed that the reduction of ASPH expression in TC-1/dASPH cells inhibited EMT in part by decreasing Snail and vimentin expression as compared to parental TC-1 cells.

### ASPH inhibition downregulates expression of Ly6 family members

**Figure 6:**
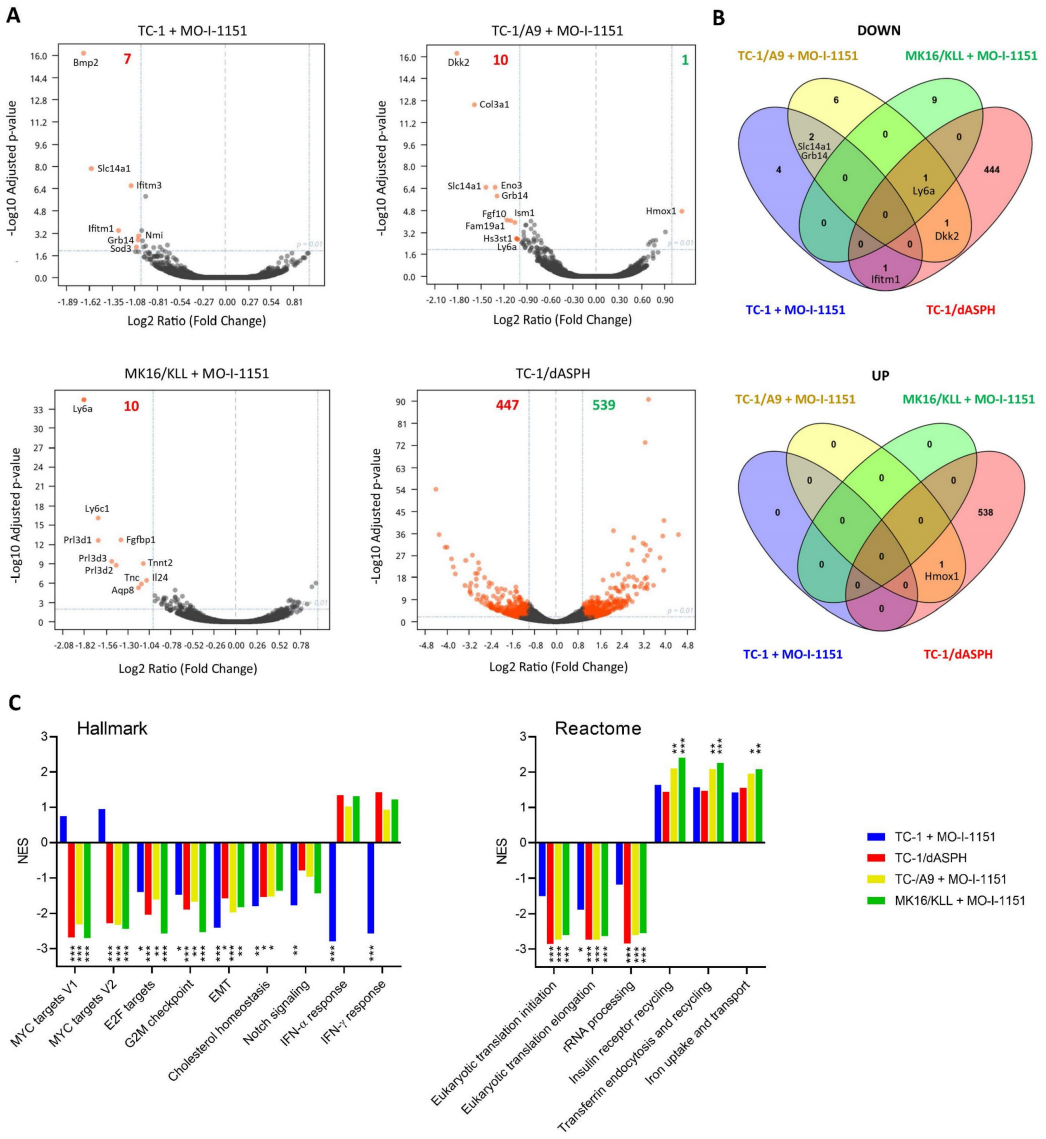
The effect of ASPH downregulation on gene expression. Bulk RNA-seq was performed after ASPH inhibition with 20 μM MO-I-1151 inhibitor for 24 h in TC-1, TC-1/A9, and MK16/KLL cells or ASPH deactivation with CRISPR/Cas9 in TC-1/dASPH cells (n=3). DMSO was used as a control**. (A)** Differential gene expression. Orange dots indicate genes with at least 2-fold decreased or increased expression and p_adj_ ≤0.01. Numbers of these genes are indicated in green and red colors, respectively. **(B)** Overlap of down- and upregulated genes. **(C)** Enrichment analysis. Some of the most significant differences found with Hallmark and Reactome gene sets are shown.

Since the effect of ASPH on the Notch1 or SRC pathways was not unambiguously shown in this study, we hypothesized that ASPH overexpression might regulate oncogenicity of the analyzed cell lines *via* another pathway. Therefore, we performed bulk RNA sequencing and determined differential gene expression (Figure 6A). After 24-hour incubation with the MO-I-1151 inhibitor, 7, 10, and 10 genes were downregulated in TC-1, TC-1/A9, and MK16/KLL cells, respectively. Only one gene (*Hmox1*) was upregulated in TC-1/A9 cells. In TC-1/dASPH cells, 447 genes were downregulated and 539 upregulated in comparison with parental TC-1 cells. Overlap analysis showed (Figure 6B), that expression of no gene was altered in all 4 cell lines analyzed and only 1 gene (*Ly6a*) was downregulated in 3 cell lines (TC-1/A9, MK16/KLL, and TC-1/dASPH) after reduction of ASPH enzymatic activity. Interferon induced transmembrane protein 1 (*Ifitm1*)*, Slc14a1, Grb14, Dkk2, and Hmox1* were down- or upregulated in two cell lines.

Analysis of the RNA-seq reads also showed the complete absence of exon 6 39 in the TC-1/dASPH transcriptome, resulting in the deletion of 38 amino acids in the ASPH protein, which could reduce the enzymatic activity of ASPH.

Enrichment analysis with preranked GSEA and hallmark gene sets (Figure 6C) revealed significant downregulation for E2F targets, G2M checkpoint, and EMT in all 4 cell lines. Lower downregulation was also found for cholesterol homeostasis and Notch signaling. Surprisingly, while Myc targets were downregulated in TC-1/A9, TC-1/dASPH, and MK16/KLL cells, this effect was not detected in TC-1 cells, where IFN signaling was highly downregulated. Further analysis of gene sets for canonical pathways (Table S1) suggested, for instance, a reduction of proteosynthesis (this effect was lower in TC-1 cells) and upregulation of processes associated with endocytosis and receptor-mediated uptake and signaling (Figure 6C).

**Figure 7:**
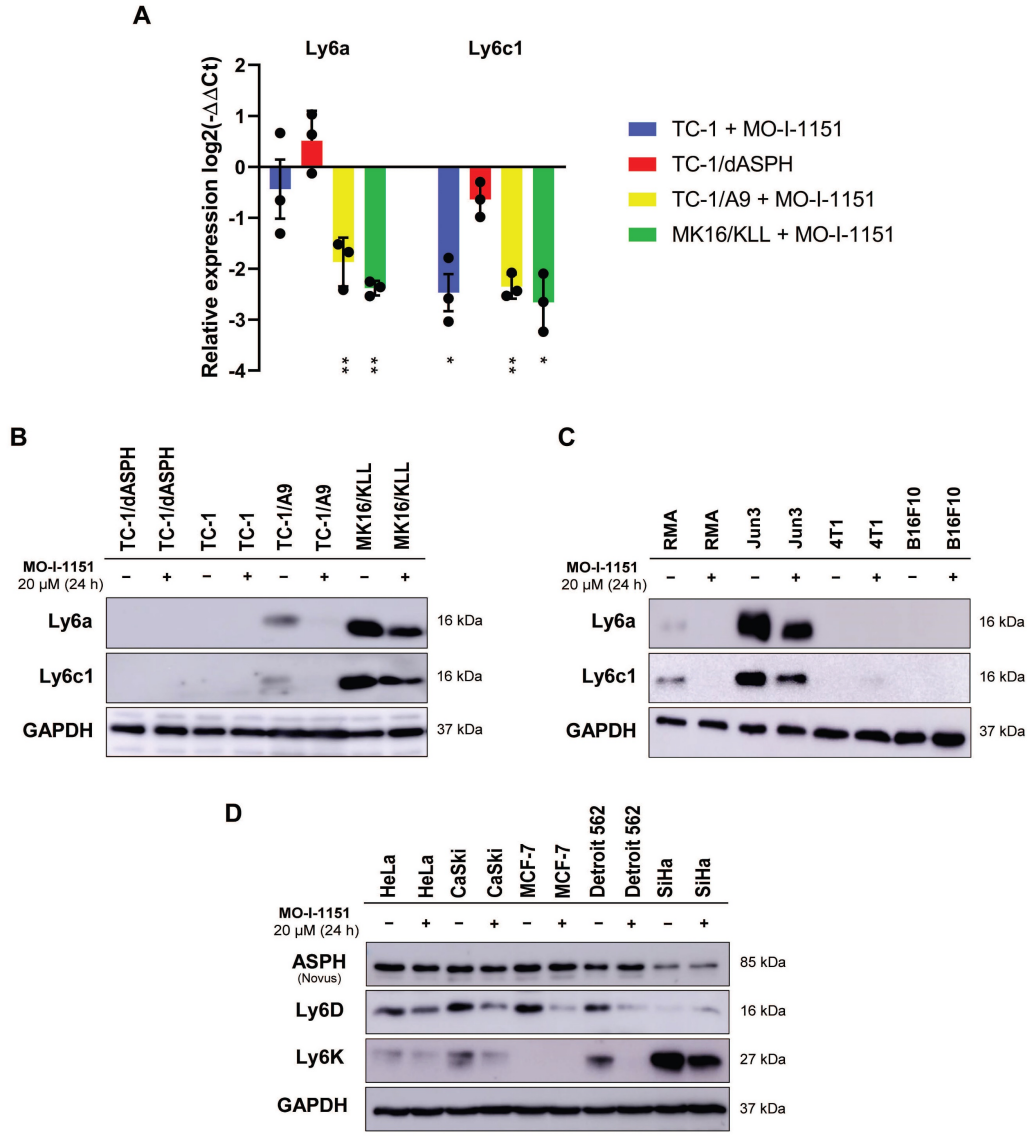
The effect of ASPH inhibition on regulation of Ly6 family members. Cells were treated with 20 μM MO-I-1151 inhibitor for 24 h and DMSO was used as a control. **(A)** *Ly6a* and *Ly6c1* expression was detected by RT-qPCR and relative quantification was calculated. Data represents the mean ± SEM of three independent experiments. TC-1, TC-1/A9, and MK16/KLL cell lines incubated with MO-I-1151 were compared with DMSO-treated controls and TC-1/dASPH cells with TC-1 cell line. * *p*<0.05, ** *p*<0.01 by *t*-test. **(B, C, D)** Ly6 proteins were examined by immunoblotting. Equal amounts of proteins were subjected to SDS-PAGE. GAPDH was used as an internal control. Ly6a and Ly6c were detected in mouse cell lines TC-1/dASPH, TC-1, TC-1/A9, and MK16/KLL **(B)** and RMA, JUN-3, 4T1, and B16-F10 **(C)** and Ly6D and Ly6K in human cell lines HeLa, CaSki, MCF-7, Detroit 562 and SiHa **(D)**.

The differential expression of the main genes identified as altered after MO-I-1151 treatment was verified by RT-qPCR. This analysis confirmed downregulation of *Ly6a* and *Ly6c1* in MK16/KLL and TC-1/A9 cells (Figure 7A) and very low expression in TC-1 and TC-1/dASPH cells (on average, 0.2-2.5 RPM in RNA-seq and Ct>33 in RT-qPCR). The levels of the Ly6 proteins corresponded to mRNA expression, when high levels of Ly6a and Ly6c were found in MK16/KLL cells, low levels in TC-1/A9 cells, and TC-1 and TC-1/dASPH cells were negative. MO-I-1151 treatment reduced Ly6a and Ly6c in MK16/KLL and TC-1/A9 cell lines (Figure 7B). RT-qPCR also confirmed the results of RNA-seq for the other tested genes. Surprisingly, while ASPH inhibition downregulated *Slc14a1* expression in TC-1 and TC-1/A9 cells, this gene was upregulated in TC-1/dASPH cells (Figure S2). MO-I-treatment downregulated *Slc14a1* expression in TC-1/dASPH cells to the level detected in TC-1 cell line.

We then tested the effect of ASPH inhibition on Ly6a and Ly6c production in four other mouse tumor cell lines (Figure 7C). We found low levels of the Ly6a and Ly6c proteins in lymphoma RMA cell line and high levels in fibrosarcoma JUN-3 cells. ASPH inhibition reduced their production in both cell lines. In melanoma B16-F10 and breast cancer 4T1 cell lines, Ly6a and Ly6c were not determined.

Finally, the quantification of two members of the Ly6 family - Ly6D and Ly6K - was performed in 5 human tumor cell lines. In HeLa, CaSki, and Detroit 562 cells, both proteins were detected, and their production was reduced after ASPH inhibition. In SiHa cell line, Ly6K was highly produced and ASPH inhibition decreased its level, but the Ly6D production was low and the effect of ASPH inhibition was not demonstrated. In MCF-7 cells, only Ly6D was detected, and its production was reduced by ASPH inhibition.

## Discussion

Since the ASPH enzyme has been identified as an important player in tumorigenesis, increased efforts are being devoted to deciphering its mechanisms of action. Notch1, SRC, vimentin, GSK-3β, and pRb signaling have already been revealed as ASPH-regulated pathways 14. However, because ASPH hydroxylates EGF-like domains that are present in more than a hundred proteins with various functions (https://en.wikipedia.org/wiki/EGF-like_domain), its involvement in malignant cell transformation, tumor progression, and escape from immune surveillance may be much broader. In this study, we attempted to find additional pathways affected by ASPH in mouse tumor cell lines using bulk RNA-seq.

For this analysis, we chose the cell lines TC-1^26^, TC-1/A9 27, and MK16/KLL 28 that we use for examination of cancer immunotherapy. Besides the origin of cells (lung *versus* kidney), TC-1 and MK16/KLL cells differ in their shape (fibroblastoid *versus* epithelioid) and an ability to form lung metastases (experimental *versus* spontaneous). In TC-1/A9 cells, epigenetic reprogramming is responsible for modification of the expression of immune-related genes 40,41, but its impact can be miscellaneous.

First, we verified ASPH synthesis in the cell lines by immunoblotting. This analysis showed markedly lower level of the ASPH protein in MK16/KLL cells. Then, we used the CRISPR/Cas9 system to deactivate the *ASPH* gene in TC-1 cells (with high endogenous ASPH level). After repeated attempts, we obtained a slowly proliferating clone TC-1/dASPH with highly reduced levels of ASPH isoforms. High-molecular-weight isoforms detected by the Boster antibody also showed a shift in the protein size corresponding to the deletion of the exon 6 found by RNA-seq. The reduced expression of ASPH observed in our study corresponds to ASPH deactivation in AsPC-1 8, MDA-MB-231 42, SGC7901 and BGC823 43 cells where a weak ASPH band was also found by immunoblotting. It also suggests an indispensable role of ASPH in cell proliferation because residual ASPH was probably necessary to obtain a proliferating clone with ASPH reduction.

To study the role of ASPH in the cell lines, we inhibited its enzymatic activity with the second-generation inhibitor MO-I-1151 and showed the effect on cell proliferation, migration, and invasion, which corresponded to the findings of the previous studies 6,19,44. We suppose that the effect of the MO-I-1151 inhibitor on cell behavior was not associated with non-specific cytotoxicity and cell death, because we did not observe cytotoxicity in long-term exposure to MO-I-1151. Notably, we did not observe any visible changes in cell morphology, and the MTT assay revealed no significant difference between long-term treatment and 48 h exposure to the MO-I-1151 inhibitor (data not shown). In addition, previous studies using MO-I-1151 and MO-I-1144 (both second-generation inhibitors) at concentrations up to 20-40 μM did not also report any cytotoxic effect 7,45. Accordingly, we assume that the effect of MO-I-1151 treatment on TC-1/dASPH cells was mediated by residual ASPH production and enzymatic activity.

Despite the increased level of ASPH in TC-1/A9 cells and decreased in MK16/KLL cells in comparison with TC-1 cells, in immunoblotting analysis of Notch1 and SRC pathways, TC-1/A9 more resembled MK16/KLL than TC-1 cells, when Notch1 signaling was more active in TC-1 cells and SRC signaling in TC-1/A9 and MK16/KLL cells. Furthermore, ASPH inhibition downregulated both total and phosphorylated c-Myc in TC-1/A9 and MK16/KLL cells but upregulated in TC-1 cells. Bulk RNA-seq analysis confirmed downregulation of genes associated with cell proliferation (gene sets E2F targets, G2M checkpoint) and EMT after ASPH inhibition, but it also showed some differences in TC-1 cells in comparison with the other cells. First, while Myc targets were highly downregulated in TC-1/dASPH, TC-1/A9, and MK16/KLL cell lines, they were rather upregulated in TC-1 cells, which corresponded to the detection of c-Myc by immunoblotting. Next, IFN-α and IFN-γ responses were strongly downregulated in TC-1 cells, but they were slightly upregulated in the other cell lines. Finally, downregulation of translational machinery was lower in TC-1 cells. The reasons for these differences are not clear but could be short-term effects (because they are not present in TC-1/dASPH cells) and/or associated with epigenetic regulation of gene expression (because in both TC-1/A9 and MK16/KLL cells, the antigen processing and presentation machinery is reversibly downregulated, which may suggest a similar epigenetic pattern).

While deactivation of *ASPH* in TC-1/dASPH cells up- or downregulated almost a thousand genes, short-term inhibition of ASPH with the MO-I-1151 inhibitor altered the expression of low numbers (7-11) of genes in TC-1, TC-1/A9, and MK16/KLL cell lines. Overlap analysis showed that no gene was dysregulated in all 4 cell lines and only 1 gene - *Ly6a* - was downregulated in 3 cell lines. This gene attracted our attention because it encodes the stem cell antigen-1 (Sca-1), which is used as a marker of mouse cancer stem cells. The human ortholog of *Ly6a* has only recently been identified 46. Ly6a/Sca-1 production was inhibited with TGF-β in mouse cancer cells 47,48. Conversely, TGF-β signaling was reduced with Ly6a/Sca-1 *via* its binding to the TGF-β receptor I and disrupting the TGF-β ligand growth differentiation factor 10 (GDF10) signaling 49. This negative feedback loop could be affected with ASPH because cbEGF-like domains are present in latent TGF-β binding proteins and fibrillins that can influence a release of the active TGF-β. Regulation of the TGF-β pathway with Ly6 proteins in human cells has been shown for Ly6E/K in breast cancer cells 50.

The family of *Ly6* genes comprises more than 30 human and 60 mouse members 51. Some of them are associated with cancer progression and poor prognosis 52,53. Besides *Ly6a*, another member of the *Ly6* family - *Ly6c1* - was significantly reduced in MK16/KLL cells by ASPH inhibition. Immunoblotting analysis confirmed downregulation of both Ly6a/Sca-1 and Ly6c proteins in these cells and showed low levels of these proteins in TC-1/A9 cells, which were decreased with the MO-I-1151 inhibitor. Ly6c is expressed on various types of mouse immune cells but its expression on solid tumor cells is not common and a human ortholog is not known 53. To extend our findings to human cells, we tested the levels of Ly6D and Ly6K proteins, which are upregulated in various tumor types 53, in 5 human tumor cell lines (HeLa, SiHa, CaSki, Detroit 562, and MCF-7) and showed their reduction after ASPH inhibition. Recently, knockdown of Ly6K expression in HeLa and SiHa cells inhibited proliferation induced by epidermal growth factor and suppressed migration and invasion stimulated by TGF-β signaling 54. In addition, Ly6K has been used as a therapeutic target in several clinical trials 55.

Brewitz *et al*. redefined the consensus sequence for ASPH hydroxylation in EGF-like domains and localized the preferred hydroxylation site to a disulfide-bridged macrocycle formed of 10 amino acid residues 56. In addition, they identified this hydroxylation site in the LYPD6B member of the LY6/urokinase-type plasminogen activator receptor superfamily, which does not contain EGF-like domains, and demonstrated ASPH-catalyzed hydroxylation of LYPD6B *in vitro*. We did not find this hydroxylation site in the LY6 proteins that we tested in our study, but because these proteins are disulfide-rich, we cannot exclude another hydroxylation site that could affect protein stability after modification with ASPH.

Among the identified genes that are differentially expressed after inhibition of ASPH, there are other genes that may be involved in carcinogenesis and/or contribute to tumor development. Grb14 is an adaptor protein with the C-terminal Src-homology 2 (SH2) domain that regulates cell growth, migration, and metabolism 57. In hepatocytes, Grb14 repressed cell division and its downregulation was found in hepatocellular carcinoma 58. Overexpression of Grb14 was associated with better overall survival of patients with breast cancer 59, but predicted poor prognosis for patients with colorectal carcinoma 60. IFN-induced transmembrane proteins, including Ifitm1, inhibit virus entry into cells and regulate both innate and adaptive immunity. Their overexpression in various cancers is a biomarker of poor prognosis, but the mechanisms involved in tumor progression are not sufficiently uncovered 61. *Slc14A1* encodes type-B urea transporter (UT-B). In different types of human tumors, Slc14A1 has been described as a tumor suppressor 62-64. Inhibition of mammalian target of rapamycin (mTOR) signaling and epigenetic regulation of oncometabolite genes have been identified as mechanisms of the Slc14A1-induced antitumor effect 62. Dkk2 regulates cell proliferation *via* Wingless-related integration site (Wnt) signaling. Its expression is upregulated in Ewing sarcoma, colorectal cancer, and pancreatic carcinoma, but downregulated in renal, ovarian, and gastrointestinal cancers 65. Hmox1 supports angiogenesis in tumors and contributes to the protection of cancer cells under increased oxidative stress 66. It can also mediate the induction of ferroptosis by cyclophosphamide treatment 67.

## Conclusions

ASPH stimulates cell proliferation, migration, and invasiveness. Several pathways regulated with this enzyme have already been revealed, but other potential targets of ASPH hydroxylation suggest that cellular functions of ASPH can be more diverse. We found two members of the *Ly6* family, *Ly6a* and *Ly6c1,* among the genes with differential expression induced by inhibition or deactivation of ASPH in tumor cells. A link with TGF-β signaling was proposed for both mouse and human Ly6 proteins, but further studies are needed to verify the possible mechanism of TGF-β cooperation with ASPH in regulating *Ly6* expression. Since increased expression of *Ly6* genes is associated with progression of both mouse and human tumors, their upregulation with ASPH can enhance the effect of ASPH in tumorigenesis and strengthen its role as a target in cancer therapy.

## Supplementary Material

Supplementary figure and table.

## Abbreviations

ADAM12/15: A disintegrin and metallopeptidase domain 12/15
ASPH: Aspartate β-hydroxylase
c-MYC: c-myelocytomatosis oncogene
Dkk2: Dickkopf WNT signaling pathway inhibitor 2
DMEM: Dulbecco's modified Eagle's medium
DMSO: Dimethyl sulfoxide
EMEM: Eagle's minimum essential medium
EMT: Epithelial-mesenchymal transition
ERK: Extracellular signal-regulated kinase
FBS: Fetal bovine serum
GAPDH: Glyceraldehyde-3-phosphate dehydrogenase
GDF10: Growth differentiation factor 10
Grb14: Growth factor receptor bound protein 14
GSEA: Gene set enrichment analysis
GSK-3β: Glycogen synthase kinase-3β
HES1: Hes family bHLH transcription factor 1
HEY1: HES with YRPW motif 1
Hmox1: Heme oxygenase 1
HPV-16: Human papillomavirus type 16
HRP: Horseradish peroxidase
Ifitm1: Interferon induced transmembrane protein 1
IFN: Interferon
Ly6: Lymphocyte antigen 6
MAPK: Mitogen-activated protein kinase
MHC-I: Major histocompatibility complex class I
MMP: Matrix metallopeptidase
MTT: 3-[4,5-dimethylthiazol-2-yl]-2,5-diphenyl-tetrazolium bromide
PBS: Phosphate-buffered saline
PCNA: Proliferating cell nuclear antigen
PI3K/Akt: Phosphatidylinositol-3-kinase/protein kinase B
pRb: Retinoblastoma protein
PVDF: Polyvinylidene difluoride
RAF: Rapidly accelerated fibrosarcoma
RAS: Rat sarcoma
RPMI-1640: Roswell Park Memorial Institute 1640
RT-qPCR: Reverse transcription quantitative polymerase chain reaction
Sca-1: Stem cell antigen-1
SDS: Sodium dodecyl-sulfate
SH2: Src-homology 2
Slc14a1: Solute carrier family 14 member 1
SMI: Small molecule inhibitor
SRC: SRC tyrosine kinase
TGF-β: Transforming growth factor beta
WNT: Wingless-related integration site
