# Supplementary material for: Aspartate β-hydroxylase Regulates Expression of Ly6 Genes
Source: J Cancer. 2024 Jan 1;15(5):1138–52. doi: 10.7150/jca.90422 (PMC10861829; doi:10.7150/jca.90422)
Supplement: Supplementary file 1 — Supplementary figure and table. [file jcav15p1138s1.pdf]

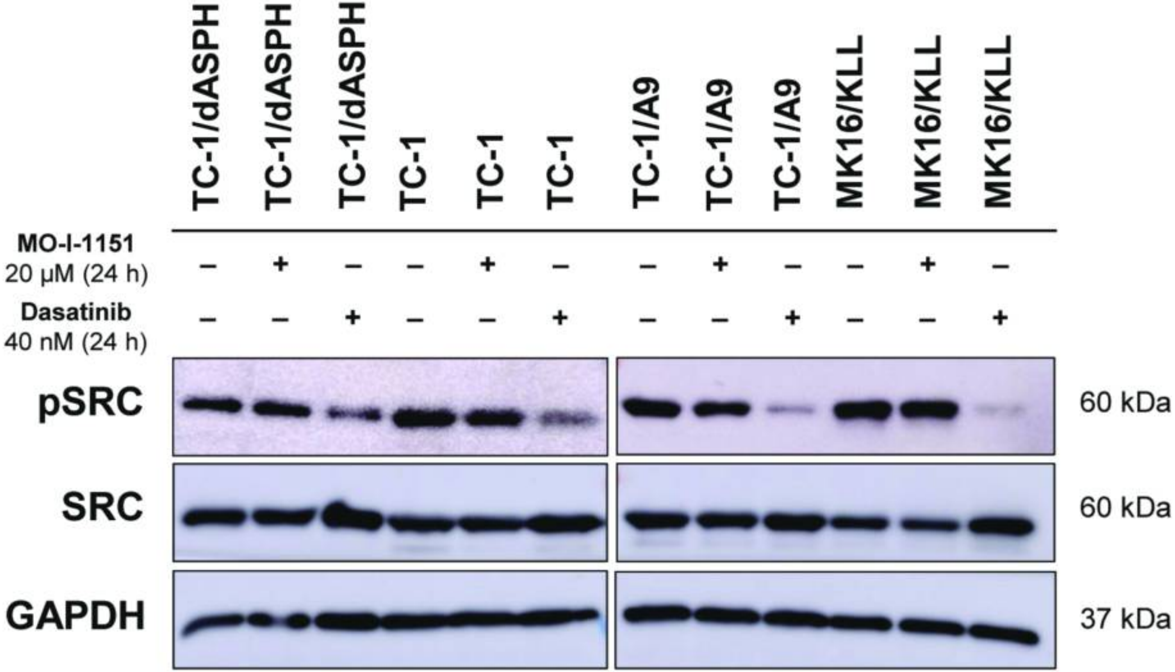

Figure S1: Immunoblotting analysis of SRC signaling. TC-1, TC-1/dASPH, TC-1/A9 and MK16/KLL cells were treated with the ASPH inhibitor (20 µM MO-I-1151) or SRC inhibitor (40 nM dasatinib) for 24 h and DMSO was used as a control. Phosphorylated SRC (pSRC) and total SRC (SRC) were detected. Glyceraldehyde-3-phosphate dehydrogenase (GAPDH) was used as an internal control.

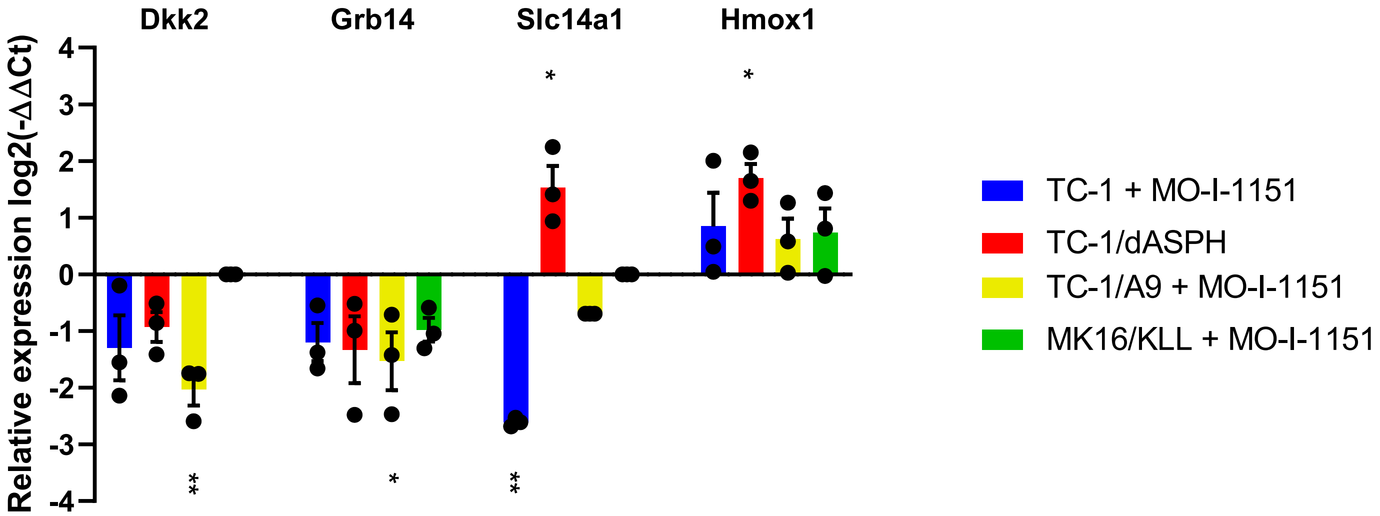

Figure S2: RT-qPCR analysis. Cells were treated with 20 μM MO-I-1151 inhibitor for 24 h and DMSO was used as a control. Expression of genes differentially expressed in RNA-seq was detected by RT-qPCR and relative quantification was calculated. Data represents the mean ± SEM of three independent experiments. TC-1, TC-1/A9, and MK16/KLL cell lines incubated with MO-I-1151 were compared with DMSO-treated controls and TC-1/dASPH cells with TC-1 cell line. * $p<0.05$, ** $p<0.01$ by t-test.

Supplementary Table 1: GSEA of canonical pathways (FDR q-val ≤ 0.05)

TC-1 + MO-I-1151

#	Gene set - downregulation	NES	FDR q-val
1	REACTOME_INTERFERON_ALPHA_BETA_SIGNALING	-2.52	0
2	PID_AVB3_INTEGRIN_PATHWAY	-2.33	0
3	REACTOME_ASSEMBLY_OF_COLLAGEN_FIBRILS_AND_OTHER_MULTIMERIC_STRUCTURES	-2.29	0
4	PID_INTEGRIN1_PATHWAY	-2.27	0
5	WP_BMP_SIGNALING_PATHWAY_IN_EYELID_DEVELOPMENT	-2.24	0
6	PID_SYNDECAN_1_PATHWAY	-2.23	0
7	REACTOME_COLLAGEN_DEGRADATION	-2.23	0
8	WP_TYPE_II_INTERFERON_SIGNALING_IFNG	-2.19	0
9	REACTOME_SYNDECAN_INTERACTIONS	-2.18	0
10	REACTOME_INTERFERON_SIGNALING	-2.15	0.001
11	WP_HEART_DEVELOPMENT	-2.13	0.001
12	PID_ANGIOPOIETIN_RECEPTOR_PATHWAY	-2.13	0.001
13	REACTOME_ACTIVATION_OF_GENE_EXPRESSION_BY_SREBF_SREBP	-2.09	0.002
14	REACTOME_ELASTIC_FIBRE_FORMATION	-2.08	0.002
15	REACTOME_SIGNALING_BY_PDGF	-2.07	0.002
16	WP_ANGIOGENESIS	-2.07	0.002
17	WP_TGFBETA_RECEPTOR_SIGNALING	-2.05	0.003
18	REACTOME_COLLAGEN_FORMATION	-2.04	0.004
19	WP_HYPOTHESIZED_PATHWAYS_IN_PATHOGENESIS_OF_CARDIOVASCULAR_DISEASE	-2.04	0.004
20	KEGG_ECM_RECEPTOR_INTERACTION	-2.04	0.004
21	REACTOME_SIGNALING_BY_PDGFR_IN_DISEASE	-2.04	0.004
22	REACTOME_NON_INTEGRIN_MEMBRANE_ECM_INTERACTIONS	-2.04	0.004
23	REACTOME_MOLECULES_ASSOCIATED_WITH_ELASTIC_FIBRES	-2.03	0.004
24	REACTOME_EXTRACELLULAR_MATRIX_ORGANIZATION	-2.03	0.004
25	PID_INTEGRIN3_PATHWAY	-2.03	0.004
26	REACTOME_CHOLESTEROL_BIOSYNTHESIS	-2.02	0.004
27	KEGG_DRUG_METABOLISM_CYTOCHROME_P450	-2.02	0.004
28	REACTOME_CROSSLINKING_OF_COLLAGEN_FIBRILS	-2.02	0.004
29	NABA_CORE_MATRISOME	-2.01	0.004
30	WP_OSTEOBLAST_DIFFERENTIATION	-2	0.005
31	PID_LYMPH_ANGIOGENESIS_PATHWAY	-2	0.005
32	REACTOME_INTERFERON_GAMMA_SIGNALING	-2	0.005
33	KEGG_MELANOMA	-2	0.005
34	REACTOME_DEGRADATION_OF_THE_EXTRACELLULAR_MATRIX	-2	0.005
35	WP_TGFBETA_RECEPTOR_SIGNALLING_IN_SKELETAL_DYSPLASIAS	-1.99	0.005
36	WP_INFLAMMATORY_RESPONSE_PATHWAY	-1.99	0.005
37	REACTOME_INTEGRIN_CELL_SURFACE_INTERACTIONS	-1.99	0.005
38	WP_MIR5093P_ALTERATION_OF_YAP1ECM_AXIS	-1.99	0.005
39	REACTOME_SCAVENGING_BY_CLASS_A_RECEPTORS	-1.99	0.005
40	WP_DIFFERENTIATION_PATHWAY	-1.96	0.007
41	NABA_COLLAGENS	-1.96	0.007
42	KEGG_PANCREATIC_CANCER	-1.96	0.006
43	WP_MIRNA_TARGETS_IN_ECM_AND_MEMBRANE_RECEPTORS	-1.96	0.006
44	NABA_ECM_GLYCOPROTEINS	-1.96	0.006
45	REACTOME_BINDING_AND_UPTAKE_OF_LIGANDS_BY_SCAVENGER_RECEPTORS	-1.95	0.007
46	WP_PI3KAKT_SIGNALING_PATHWAY	-1.95	0.007
47	WP_THE_HUMAN_IMMUNE_RESPONSE_TO_TUBERCULOSIS	-1.95	0.007
48	REACTOME_REGULATION_OF_CHOLESTEROL_BIOSYNTHESIS_BY_SREBP_SREBF	-1.94	0.008
49	PID_VEGFR1_PATHWAY	-1.94	0.008
50	WP_ADIPOGENESIS	-1.94	0.008
51	REACTOME_TIE2_SIGNALING	-1.93	0.009
52	WP_OLIGODENDROCYTE_SPECIFICATION_AND_DIFFERENTIATION_LEADING_TO_MYELIN_COMPONENTS_FOR_CNS	-1.92	0.009
53	WP_HAIR_FOLLICLE_DEVELOPMENT_ORGANOGENESIS_PART_2_OF_3	-1.92	0.009
54	REACTOME_NOTCH4_INTRACELLULAR_DOMAIN_REGULATES_TRANSCRIPTION	-1.92	0.009
55	REACTOME_SRP_DEPENDENT_COTRANSLATIONAL_PROTEIN_TARGETING_TO_MEMBRANE	-1.92	0.009
56	REACTOME_COLLAGEN_CHAIN_TRIMERIZATION	-1.92	0.009
57	REACTOME_NOTCH3_INTRACELLULAR_DOMAIN_REGULATES_TRANSCRIPTION	-1.92	0.009
58	PID_IL4_2PATHWAY	-1.92	0.009
59	KEGG_BLADDER_CANCER	-1.91	0.009

60	REACTOME_COLLAGEN_BIOSYNTHESIS_AND_MODIFYING_ENZYMES	-1.9	0.01
61	BIOCARTA_EICOSANOID_PATHWAY	-1.9	0.011
62	KEGG_METABOLISM_OF_XENOBIOTICS_BY_CYTOCHROME_P450	-1.89	0.011
63	WP_FOCAL_ADHESIONPI3KAKTMTORSIGNALING_PATHWAY	-1.89	0.012
64	WP_TGFB_SIGNALING_IN_THYROID_CELLS_FOR_EPITHELIALMESENCHYMAL_TRANSITION	-1.89	0.012
65	PID_ERBB1_RECEPTOR_PROXIMAL_PATHWAY	-1.89	0.012
66	REACTOME_EUKARYOTIC_TRANSLATION_ELONGATION	-1.88	0.012
67	WP_GLUCURONIDATION	-1.88	0.012
68	KEGG_PATHWAYS_IN_CANCER	-1.88	0.012
69	REACTOME_NICOTINAMIDE_SALVAGING	-1.87	0.013
70	REACTOME_CELL_SURFACE_INTERACTIONS_AT_THE_VASCULAR_WALL	-1.87	0.013
71	WP_CHOLESTEROL_BIOSYNTHESIS_PATHWAY	-1.87	0.014
72	REACTOME_PI_3K_CASCADE_FGFR2	-1.87	0.014
73	REACTOME_INTERLEUKIN_4_AND_INTERLEUKIN_13_SIGNALING	-1.86	0.014
74	REACTOME_INTERLEUKIN_37_SIGNALING	-1.86	0.014
75	REACTOME_O_GLYCOSYLATION_OF_TSR_DOMAIN_CONTAINING_PROTEINS	-1.86	0.014
76	REACTOME_YAP1_AND_WWTR1_TAZ_STIMULATED_GENE_EXPRESSION	-1.86	0.014
77	WP_CARDIAC_PROGENITOR_DIFFERENTIATION	-1.86	0.014
78	WP_HAIR_FOLLICLE_DEVELOPMENT_CYTODIFFERENTIATION_PART_3_OF_3	-1.85	0.015
79	WP_FGFR3_SIGNALLING_IN_CHONDROCYTE_PROLIFERATION_AND_TERMINAL_DIFFERENTIATION	-1.85	0.015
80	KEGG_GLIOMA	-1.85	0.015
81	REACTOME_TRANSCRIPTIONAL_REGULATION_BY_RUNX2	-1.85	0.015
82	KEGG_RIBOSOME	-1.85	0.015
83	REACTOME_RUNX2_REGULATES_BONE_DEVELOPMENT	-1.85	0.015
84	PID_AR_PATHWAY	-1.85	0.015
85	WP_EPITHELIAL_TO_MESENCHYMAL_TRANSITION_IN_COLORECTAL_CANCER	-1.84	0.015
86	WP_IL7_SIGNALING_PATHWAY	-1.84	0.017
87	PID_TCPTP_PATHWAY	-1.83	0.018
88	REACTOME_DISEASES_ASSOCIATED_WITH_O_GLYCOSYLATION_OF_PROTEINS	-1.83	0.018
89	WP_SARSCOV2_INNATE_IMMUNITY_EVASION_AND_CELLSPECIFIC_IMMUNE_RESPONSE	-1.83	0.018
90	REACTOME_DOWNSTREAM_SIGNALING_OF_ACTIVATED_FGFR2	-1.83	0.018
91	REACTOME_MET_PROMOTES_CELL_MOTILITY	-1.82	0.019
92	REACTOME_NCAM1_INTERACTIONS	-1.82	0.019
93	REACTOME_NICOTINATE_METABOLISM	-1.82	0.019
94	PID_DELTA_NP63_PATHWAY	-1.81	0.022
95	WP_CYTOPLASMIC_RIBOSOMAL_PROTEINS	-1.81	0.023
96	REACTOME_SIGNALING_BY_CYTOSOLIC_FGFR1_FUSION_MUTANTS	-1.81	0.022
97	WP_TYPE_I_COLLAGEN_SYNTHESIS_IN_THE_CONTEXT_OF_OSTEOGENESIS_IMPERFECTA	-1.8	0.023
98	WP_SARS_CORONAVIRUS_AND_INNATE_IMMUNITY	-1.8	0.023
99	WP_ENDOTHELIN_PATHWAYS	-1.8	0.023
100	KEGG_HEDGEHOG_SIGNALING_PATHWAY	-1.8	0.024
101	WP_MFAP5_EFFECT_ON_PERMEABILITY_AND_MOTILITY_OF_ENDOTHELIAL_CELLS_VIA_CYTOSKELETON_REARRANGEMENT	-1.79	0.025
102	PID_ERBB4_PATHWAY	-1.79	0.026
103	WP_RAC1PAK1P38MMP2_PATHWAY	-1.79	0.026
104	WP_SENESCENCE_AND_AUTOPHAGY_IN_CANCER	-1.78	0.026
105	REACTOME_CONSTITUTIVE_SIGNALING_BY_ABERRANT_PI3K_IN_CANCER	-1.78	0.026
106	REACTOME_INTERLEUKIN_20_FAMILY_SIGNALING	-1.78	0.028
107	WP_SMALL_LIGAND_GPCRS	-1.78	0.029
108	WP_INTERFERON_TYPE_I_SIGNALING_PATHWAYS	-1.78	0.028
109	NABA_SECRETED_FACTORS	-1.77	0.028
110	WP_CANONICAL_AND_NONCANONICAL_NOTCH_SIGNALING	-1.77	0.028
111	REACTOME_MET_ACTIVATES_PTK2_SIGNALING	-1.77	0.028
112	WP_SIGNALING_PATHWAYS_IN_GLIOBLASTOMA	-1.77	0.028
113	REACTOME_REGULATION_OF_BETA_CELL_DEVELOPMENT	-1.77	0.029
114	REACTOME_ECM_PROTEOGLYCANS	-1.77	0.029
115	WP_MESODERMAL_COMMITMENT_PATHWAY	-1.76	0.031
116	PID_MYC_REPRESS_PATHWAY	-1.76	0.031
117	REACTOME_DOWNSTREAM_SIGNAL_TRANSDUCTION	-1.76	0.032
118	WP_EGFR_TYROSINE_KINASE_INHIBITOR_RESISTANCE	-1.75	0.033
119	WP_AGERAGE_PATHWAY	-1.75	0.034
120	PID_BMP_PATHWAY	-1.75	0.035
121	WP_CHOLESTEROL_METABOLISM_INCLUDES_BOTH_BLOCH_AND_KANDUTSCHRUSSELL_PATHWAYS	-1.74	0.035
122	WP_RETINOBLASTOMA_GENE_IN_CANCER	-1.74	0.037
123	KEGG_FOCAL_ADHESION	-1.74	0.036

124	REACTOME_ANCHORING_FIBRIL_FORMATION	-1.73	0.039
125	WP_PHOTODYNAMIC_THERAPYINDUCED_AP1_SURVIVAL_SIGNALING	-1.73	0.04
126	REACTOME_RUNX2_REGULATES_OSTEOBLAST_DIFFERENTIATION	-1.72	0.044
127	WP_PROSTAGLANDIN_SYNTHESIS_AND_REGULATION	-1.72	0.045
128	WP_PDGFRBETA_PATHWAY	-1.71	0.047
129	WP_HEPATITIS_B_INFECTION	-1.71	0.047
130	KEGG_BASAL_CELL_CARCINOMA	-1.71	0.047
131	WP_CANCER_IMMUNOTHERAPY_BY_PD1_BLOCKADE	-1.71	0.047
132	REACTOME_REGULATION_OF_INSULIN_LIKE_GROWTH_FACTOR_IGF_TRANSPORT_AND_UPTAKE_BY_INSULIN_LIKE_GROWTH_FACTOR_BINDING_PROTEINS_IGFBPS	-1.71	0.048
133	WP_MICRORNAS_IN_CARDIOMYOCYTE_HYPERTROPHY	-1.7	0.048
134	PID_TOLL_ENDOGENOUS_PATHWAY	-1.7	0.05

TC-1/dASPH

#	Gene set - upregulation	NES	FDR q-val
1	KEGG_PENTOSE_AND_GLUCURONATE_INTERCONVERSIONS	2.12	0.01
2	WP_GLUCURONIDATION	2.09	0.01
3	KEGG_PORPHYRIN_AND_CHLOROPHYLL_METABOLISM	2.01	0.026
4	REACTOME_NITRIC_OXIDE_STIMULATES_GUANYLATE_CYCLASE	1.95	0.047
5	WP_SIMPLIFIED_DEPICTION_OF_MYD88_DISTINCT_INPUTOUTPUT_PATHWAY	1.93	0.05

#	Gene set - downregulation	NES	FDR q-val
1	REACTOME_EUKARYOTIC_TRANSLATION_INITIATION	-2.85	0
2	REACTOME_RRNA_PROCESSING	-2.84	0
3	REACTOME_NONSENSE_MEDIATED_DECAY_NMD	-2.81	0
4	KEGG_RIBOSOME	-2.78	0
5	REACTOME_EUKARYOTIC_TRANSLATION_ELONGATION	-2.73	0
6	REACTOME_TRANSLATION	-2.69	0
7	WP_CYTOPLASMIC_RIBOSOMAL_PROTEINS	-2.68	0
8	REACTOME_SRP_DEPENDENT_COTRANSLATIONAL_PROTEIN_TARGETING_TO_MEMBRANE	-2.68	0
9	REACTOME_INFLUENZA_INFECTION	-2.6	0
10	REACTOME_SELENOAMINO_ACID_METABOLISM	-2.59	0
11	REACTOME_ACTIVATION_OF_THE_MRNA_UPON_BINDING_OF_THE_CAP_BINDING_COMPLEX_AND_EIFS_AND_SUBSEQUENT_BINDING_TO_43S	-2.59	0
12	REACTOME_RESPONSE_OF_EIF2AK4_GCN2_TO_AMINO_ACID_DEFICIENCY	-2.59	0
13	REACTOME_REGULATION_OF_EXPRESSION_OF_SLITS_AND_ROBOS	-2.48	0
14	REACTOME_RRNA_MODIFICATION_IN_THE_NUCLEUS_AND_CYTOSOL	-2.33	0
15	REACTOME_THE_ROLE_OF_GTSE1_IN_G2_M_PROGRESSION_AFTER_G2_CHECKPOINT	-2.26	0
16	WP_METABOLIC_REPROGRAMMING_IN_COLON_CANCER	-2.25	0
17	REACTOME_SIGNALING_BY_ROBO_RECEPTORS	-2.24	0
18	REACTOME_COOPERATION_OF_PREFOLDIN_AND_TRIC_CCT_IN_ACTIN_AND_TUBULIN_FOLDING	-2.19	0
19	REACTOME_METABOLISM_OF_AMINO_ACIDS_AND_DERIVATIVES	-2.19	0
20	REACTOME_FORMATION_OF_TUBULIN_FOLDING_INTERMEDIATES_BY_CCT_TRIC	-2.16	0
21	REACTOME_CYTOSOLIC_TRNA_AMINOACYLATION	-2.16	0
22	WP_TRANSLATION_FACTORS	-2.15	0
23	WP_MRNA_PROCESSING	-2.12	0
24	REACTOME_NEGATIVE_REGULATION_OF_NOTCH4_SIGNALING	-2.08	0.001
25	REACTOME_AUF1_HNRNP_D0_BINDS_AND_DESTABILIZES_MRNA	-2.08	0.001
26	REACTOME_REGULATION_OF_RUNX3_EXPRESSION_AND_ACTIVITY	-2.08	0.001
27	REACTOME_GAP_JUNCTION_ASSEMBLY	-2.07	0.001
28	REACTOME_REGULATION_OF_MRNA_STABILITY_BY_PROTEINS_THAT_BIND_AU_RICH_ELEMENTS	-2.07	0.001
29	KEGG_SPLICEOSOME	-2.04	0.002
30	REACTOME_CELLULAR_RESPONSE_TO_STARVATION	-2.03	0.002
31	REACTOME_PROCESSING_OF_CAPPED_INTRON_CONTAINING_PRE_MRNA	-2.01	0.003
32	REACTOME_HOST_INTERACTIONS_OF_HIV_FACTORS	-2	0.004
33	REACTOME_MRNA_SPLICING	-1.99	0.004
34	REACTOME_RECYCLING_PATHWAY_OF_L1	-1.98	0.005
35	REACTOME_ASSEMBLY_OF_THE_PRE_REPLICATIVE_COMPLEX	-1.97	0.006
36	KEGG_PROTEASOME	-1.95	0.009
37	REACTOME_HCMV_EARLY_EVENTS	-1.91	0.014
38	REACTOME_GAP_JUNCTION_TRAFFICKING_AND_REGULATION	-1.91	0.014
39	REACTOME_MAPK6_MAPK4_SIGNALING	-1.91	0.014
40	PID_AURORA_B_PATHWAY	-1.91	0.014

41	REACTOME_MRNA_SPLICING_MINOR_PATHWAY	-1.9	0.015
42	REACTOME_RNA_POLYMERASE_I_TRANSCRIPTION_INITIATION	-1.9	0.016
43	REACTOME_NEGATIVE_EPIGENETIC_REGULATION_OF_RRNA_EXPRESSION	-1.89	0.016
44	PID_MYC_ACTIV_PATHWAY	-1.89	0.017
45	REACTOME_DOWNSTREAM_SIGNALING_EVENTS_OF_B_CELL_RECEPTOR_BCR	-1.88	0.017
46	WP_VEGFAVEGFR2_SIGNALING_PATHWAY	-1.88	0.017
47	REACTOME_DEFECTIVE_CFTR_CAUSES_CYSTIC_FIBROSIS	-1.88	0.017
48	REACTOME_TRNA_AMINOACYLATION	-1.88	0.016
49	REACTOME_UB_SPECIFIC_PROCESSING_PROTEASES	-1.88	0.016
50	REACTOME_ORC1_REMOVAL_FROM_CHROMATIN	-1.88	0.017
51	REACTOME_RNA_POLYMERASE_I_TRANSCRIPTION_TERMINATION	-1.87	0.018
52	WP_NSP1_FROM_SARSCOV2_INHIBITS_TRANSLATION_INITIATION_IN_THE_HOST_CELL	-1.86	0.02
53	REACTOME_DECTIN_1_MEDIATED_NONCANONICAL_NF_KB_SIGNALING	-1.86	0.021
54	REACTOME_HIV_INFECTION	-1.85	0.021
55	REACTOME_SNRNP_ASSEMBLY	-1.85	0.021
56	REACTOME_POSITIVE_EPIGENETIC_REGULATION_OF_RRNA_EXPRESSION	-1.85	0.023
57	REACTOME_REGULATION_OF_HMOX1_EXPRESSION_AND_ACTIVITY	-1.85	0.023
58	REACTOME_REGULATION_OF_PTEN_STABILITY_AND_ACTIVITY	-1.84	0.026
59	REACTOME_CROSS_PRESENTATION_OF_SOLUBLE_EXOGENOUS_ANTIGENS_ENDOSOMES	-1.83	0.026
60	REACTOME_TRANSCRIPTIONAL_REGULATION_BY_RUNX3	-1.83	0.027
61	REACTOME_METABOLISM_OF_POLYAMINES	-1.83	0.026
62	REACTOME_SCF_SKP2_MEDIATED_DEGRADATION_OF_P27_P21	-1.83	0.027
63	WP_PROTEASOME_DEGRADATION	-1.83	0.027
64	REACTOME_SEPARATION_OF_SISTER_CHROMATIDS	-1.82	0.028
65	REACTOME_STABILIZATION_OF_P53	-1.81	0.033
66	REACTOME_AGGREPHAGY	-1.8	0.035
67	REACTOME_MRNA_CAPPING	-1.8	0.035
68	REACTOME_RUNX1_REGULATES_TRANSCRIPTION_OF_GENES_INVOLVED_IN_DIFFERENTIATION_OF_HSCS	-1.8	0.036
69	PID_PLK1_PATHWAY	-1.8	0.036
70	PID_PI3KCI_PATHWAY	-1.79	0.037
71	REACTOME_SIGNALING_BY_THE_B_CELL_RECEPTOR_BCR	-1.79	0.037
72	REACTOME_RHO_GTPASES_ACTIVATE_IQGAPS	-1.79	0.037
73	REACTOME_DEGRADATION_OF_AXIN	-1.79	0.036
74	REACTOME_RESOLUTION_OF_SISTER_CHROMATID_COHESION	-1.79	0.036
75	REACTOME_MITOTIC_METAPHASE_AND_ANAPHASE	-1.79	0.036
76	REACTOME_RHO_GTPASES_ACTIVATE_FORMINS	-1.79	0.036
77	WP_ALPHA_6_BETA_4_SIGNALING_PATHWAY	-1.78	0.036
78	REACTOME_POST_CHAPERONIN_TUBULIN_FOLDING_PATHWAY	-1.78	0.037
79	REACTOME_SEALING_OF_THE_NUCLEAR_ENVELOPE_NE_BY_ESCRT_III	-1.78	0.037
80	REACTOME_RHOH_GTPASE_CYCLE	-1.78	0.037
81	REACTOME_CLEC7A_DECTIN_1_SIGNALING	-1.78	0.036
82	REACTOME_FORMATION_OF_THE_CORNIFIED_ENVELOPE	-1.78	0.036
83	REACTOME_DEGRADATION_OF_GLI1_BY_THE_PROTEASOME	-1.78	0.036
84	REACTOME_KERATINIZATION	-1.78	0.036
85	REACTOME_B_WICH_COMPLEX_POSITIVELY_REGULATES_RRNA_EXPRESSION	-1.78	0.036
86	REACTOME_CELLULAR_RESPONSE_TO_HYPOXIA	-1.77	0.037
87	REACTOME_DNA_REPLICATION_PRE_INITIATION	-1.77	0.037
88	REACTOME_HCMV_INFECTION	-1.77	0.037
89	REACTOME_LAMININ_INTERACTIONS	-1.77	0.037
90	REACTOME_HDACS_DEACETYLATE_HISTONES	-1.77	0.038
91	REACTOME_REGULATION_OF_RAS_BY_GAPS	-1.77	0.038
92	REACTOME_MITOTIC_G2_G2_M_PHASES	-1.76	0.038
93	REACTOME_DEADENYLATION_DEPENDENT_MRNA_DECAY	-1.75	0.043
94	REACTOME_EPIGENETIC_REGULATION_OF_GENE_EXPRESSION	-1.75	0.044
95	REACTOME_PCP_CE_PATHWAY	-1.75	0.045
96	REACTOME_RNA_POLYMERASE_I_TRANSCRIPTION	-1.74	0.046
97	REACTOME_G1_S_DNA_DAMAGE_CHECKPOINTS	-1.74	0.046
98	REACTOME_RNA_POLYMERASE_I_PROMOTER_ESCAPE	-1.73	0.05

TC-1/A9 + MO-I-1151

#	Gene set - upregulation	NES	FDR q-val
1	KEGG_LYSOSOME	2.28	0.001
2	WP_PHOTODYNAMIC_THERAPYINDUCED_NFE2L2_NRF2_SURVIVAL_SIGNALING	2.15	0.003

3	REACTOME_INSULIN_RECEPTOR_RECYCLING	2.11	0.003
4	REACTOME_TRANSFERRIN_ENDOCYTOSIS_AND_RECYCLING	2.09	0.003
5	WP_OXIDATIVE_STRESS	2.08	0.003
6	KEGG_REGULATION_OF_AUTOPHAGY	2.06	0.005
7	KEGG_GLYCOSAMINOGLYCAN_DEGRADATION	2.04	0.005
8	REACTOME_ROS_AND_RNS_PRODUCTION_IN_PHAGOCYTES	2.03	0.005
9	WP_GLYCOSAMINOGLYCAN_DEGRADATION	1.98	0.011
10	REACTOME_IRON_UPTAKE_AND_TRANSPORT	1.96	0.013
11	WP_NRF2_PATHWAY	1.93	0.019
12	REACTOME_PURINERGIC_SIGNALING_IN_LEISHMANIASIS_INFECTION	1.92	0.021
13	REACTOME_METABOLISM_OF_PORPHYRINS	1.89	0.028
14	REACTOME_LDL_CLEARANCE	1.86	0.039
15	REACTOME_IL_6_TYPE_CYTOKINE_RECEPTOR_LIGAND_INTERACTIONS	1.84	0.045
16	BIOCARTA_NFKB_PATHWAY	1.83	0.049

#	Gene set - downregulation	NES	FDR q-val
1	REACTOME_EUKARYOTIC_TRANSLATION_INITIATION	-2.73	0
2	REACTOME_EUKARYOTIC_TRANSLATION_ELONGATION	-2.73	0
3	KEGG_RIBOSOME	-2.7	0
4	WP_CYTOPLASMIC_RIBOSOMAL_PROTEINS	-2.68	0
5	REACTOME_SRP_DEPENDENT_COTRANSLATIONAL_PROTEIN_TARGETING_TO_MEMBRANE	-2.61	0
6	REACTOME_RRNA_PROCESSING	-2.6	0
7	REACTOME_NONSENSE_MEDIATED_DECAY_NMD	-2.53	0
8	REACTOME_RESPONSE_OF_EIF2AK4_GCN2_TO_AMINO_ACID_DEFICIENCY	-2.46	0
9	REACTOME_ACTIVATION_OF_THE_MRNA_UPON_BINDING_OF_THE_CAP_BINDING_COMPLEX_AND_EIFS_AND_SUBSEQUENT_BINDING_TO_43S	-2.43	0
10	REACTOME_SELENOAMINO_ACID_METABOLISM	-2.39	0
11	REACTOME_ASSEMBLY_OF_COLLAGEN_FIBRILS_AND_OTHER_MULTIMERIC_STRUCTURES	-2.36	0
12	REACTOME_INFLUENZA_INFECTION	-2.31	0
13	REACTOME_TRANSLATION	-2.27	0
14	WP_INFLAMMATORY_RESPONSE_PATHWAY	-2.24	0
15	REACTOME_MET_ACTIVATES_PTK2_SIGNALING	-2.2	0
16	REACTOME_SCAVENGING_BY_CLASS_A_RECEPTORS	-2.17	0
17	REACTOME_REGULATION_OF_EXPRESSION_OF_SLITS_AND_ROBOS	-2.14	0.001
18	PID_INTEGRIN1_PATHWAY	-2.08	0.002
19	REACTOME_SIGNALING_BY_ROBO_RECEPTORS	-2.08	0.002
20	PID_AVB3_INTEGRIN_PATHWAY	-2.08	0.002
21	NABA_COLLAGENS	-2.05	0.003
22	REACTOME_CROSSLINKING_OF_COLLAGEN_FIBRILS	-2.05	0.002
23	REACTOME_MET_PROMOTES_CELL_MOTILITY	-2.04	0.003
24	WP_MIRNA_TARGETS_IN_ECM_AND_MEMBRANE_RECEPTORS	-2.03	0.003
25	PID_SYNDECAN_1_PATHWAY	-2.02	0.004
26	PID_AR_PATHWAY	-2.02	0.004
27	REACTOME_COLLAGEN_CHAIN_TRIMERIZATION	-2.01	0.004
28	REACTOME_RRNA_MODIFICATION_IN_THE_NUCLEUS_AND_CYTOSOL	-2	0.005
29	WP_MIR5093P_ALTERATION_OF_YAP1ECM_AXIS	-1.99	0.005
30	REACTOME_COLLAGEN_FORMATION	-1.98	0.006
31	REACTOME_RNA_POLYMERASE_I_TRANSCRIPTION_INITIATION	-1.98	0.006
32	WP_GDNFRET_SIGNALLING_AXIS	-1.92	0.013
33	PID_BMP_PATHWAY	-1.92	0.013
34	REACTOME_O_GLYCOSYLATION_OF_TSR_DOMAIN_CONTAINING_PROTEINS	-1.91	0.015
35	NABA_CORE_MATRISOME	-1.89	0.017
36	REACTOME_BINDING_AND_UPTAKE_OF_LIGANDS_BY_SCAVENGER_RECEPTORS	-1.87	0.022
37	WP_NCRNAS_INVOLVED_IN_WNT_SIGNALING_IN_HEPATOCELLULAR_CARCINOMA	-1.87	0.021
38	WP_BMP_SIGNALING_PATHWAY_IN_EYELID_DEVELOPMENT	-1.87	0.021
39	REACTOME_COLLAGEN_DEGRADATION	-1.86	0.022
40	KEGG_ECM_RECEPTOR_INTERACTION	-1.85	0.028
41	KEGG_FOCAL_ADHESION	-1.85	0.027
42	REACTOME_ANCHORING_FIBRIL_FORMATION	-1.84	0.028
43	KEGG_MELANOMA	-1.84	0.028
44	WP_HAIR_FOLLICLE_DEVELOPMENT_ORGANOGENESIS_PART_2_OF_3	-1.84	0.028
45	REACTOME_NON_INTEGRIN_MEMBRANE_ECM_INTERACTIONS	-1.83	0.028
46	WP_FOCAL_ADHESIONPI3KAKTMTORSIGNALING_PATHWAY	-1.83	0.029
47	REACTOME_DEGRADATION_OF_THE_EXTRACELLULAR_MATRIX	-1.83	0.028
48	PID_MYC_ACTIV_PATHWAY	-1.83	0.028

49	REACTOME_EXTRACELLULAR_MATRIX_ORGANIZATION	-1.83	0.027
50	REACTOME_ELASTIC_FIBRE_FORMATION	-1.82	0.031
51	WP_PI3KAKT_SIGNALING_PATHWAY	-1.81	0.035
52	WP_OLIGODENDROCYTE_SPECIFICATION_AND_DIFFERENTIATION_LEADING_TO_MYELIN_COMPONENTS_FOR_CNS	-1.8	0.037
53	REACTOME_MRNA_DECAY_BY_3_TO_5_EXORIBONUCLEASE	-1.8	0.038
54	REACTOME_PLATELET_ADHESION_TO_EXPOSED_COLLAGEN	-1.79	0.042
55	REACTOME_SYNDECAN_INTERACTIONS	-1.79	0.041
56	REACTOME_COLLAGEN_BIOSYNTHESIS_AND_MODIFYING_ENZYMES	-1.78	0.044
57	REACTOME_CELLULAR_RESPONSE_TO_STARVATION	-1.78	0.045
58	REACTOME_CHOLESTEROL_BIOSYNTHESIS	-1.77	0.044
59	REACTOME_REGULATION_OF_TLR_BY_ENDOGENOUS_LIGAND	-1.77	0.044

MK16/KLL + MO-I-1151

#	Gene set - upregulation	NES	FDR q-val
1	KEGG_LYSOSOME	2.44	0
2	REACTOME_INSULIN_RECEPTOR_RECYCLING	2.41	0
3	REACTOME_TRANSFERRIN_ENDOCYTOSIS_AND_RECYCLING	2.26	0
4	REACTOME_ROS_AND_RNS_PRODUCTION_IN_PHAGOCYTES	2.22	0
5	REACTOME_AMINO_ACIDS_REGULATE_MTORC1	2.19	0
6	KEGG_EPITHELIAL_CELL_SIGNALING_IN_HELICOBACTER_PYLORI_INFECTION	2.16	0
7	KEGG_VIBRIO_CHOLERAE_INFECTION	2.16	0
8	REACTOME_IRON_UPTAKE_AND_TRANSPORT	2.08	0.002
9	REACTOME_IL_6_TYPE_CYTOKINE_RECEPTOR_LIGAND_INTERACTIONS	1.98	0.011
10	REACTOME_ASPARAGINE_N_LINKED_GLYCOSYLATION	1.97	0.011
11	REACTOME_TBC_RABGAPS	1.95	0.016
12	KEGG_GLYCOSAMINOGLYCAN_DEGRADATION	1.94	0.016
13	REACTOME_SYNTHESIS_OF_SUBSTRATES_IN_N_GLYCAN_BIOSYTHESIS	1.89	0.029
14	WP_TYPE_I_INTERFERON_INDUCTION_AND_SIGNALING_DURING_SARSCOV2_INFECTION	1.89	0.027
15	REACTOME_SIGNALING_BY_INSULIN_RECEPTOR	1.89	0.027
16	WP_HOSTPATHOGEN_INTERACTION_OF_HUMAN_CORONA_VIRUSES_AUTOPHAGY	1.88	0.03
17	REACTOME_BIOSYNTHESIS_OF_THE_N_GLYCAN_PRECURSOR_DOLICHOL_LIPID_LINKED_OLIGOSACCHARIDE_LLO_AND_TRANSFER_TO_A_NASCENT_PROTEIN	1.86	0.035
18	WP_GLYCOSAMINOGLYCAN_DEGRADATION	1.86	0.034
19	WP_HOSTPATHOGEN_INTERACTION_OF_HUMAN_CORONA_VIRUSES_INTERFERON_INDUCTION	1.86	0.034
20	REACTOME_RAB_REGULATION_OF_TRAFFICKING	1.85	0.034
21	REACTOME_NRIF_SIGNALS_CELL_DEATH_FROM_THE_NUCLEUS	1.85	0.033
22	REACTOME_INTRA_GOLGI_TRAFFIC	1.85	0.034
23	REACTOME_INTERLEUKIN_2_FAMILY_SIGNALING	1.84	0.036
24	BIOCARTA_TID_PATHWAY	1.83	0.038
25	WP_OVERVIEW_OF_INTERFERONSMEDIATED_SIGNALING_PATHWAY	1.82	0.039
26	REACTOME_P75NTR_SIGNALS_VIA_NF_KB	1.82	0.038
27	WP_NEURODEGENERATION_WITH_BRAIN_IRON_ACCUMULATION_NBIA_SUBTYPES_PATHWAY	1.82	0.038
28	BIOCARTA_CCR5_PATHWAY	1.8	0.047
29	WP_TYPE_II_INTERFERON_SIGNALING_IFNG	1.8	0.046

#	Gene set - downregulation	NES	FDR q-val
1	REACTOME_EUKARYOTIC_TRANSLATION_ELONGATION	-2.63	0
2	KEGG_RIBOSOME	-2.63	0
3	REACTOME_EUKARYOTIC_TRANSLATION_INITIATION	-2.6	0
4	REACTOME_RRNA_PROCESSING	-2.55	0
5	WP_CYTOPLASMIC_RIBOSOMAL_PROTEINS	-2.52	0
6	REACTOME_SRP_DEPENDENT_COTRANSLATIONAL_PROTEIN_TARGETING_TO_MEMBRANE	-2.5	0
7	REACTOME_NONSENSE_MEDIATED_DECAY_NMD	-2.48	0
8	REACTOME_SELENOAMINO_ACID_METABOLISM	-2.45	0
9	REACTOME_INFLUENZA_INFECTION	-2.44	0
10	REACTOME_ACTIVATION_OF_THE_MRNA_UPON_BINDING_OF_THE_CAP_BINDING_COMPLEX_AND_EIFS_AND_SUBSEQUENT_BINDING_TO_43S	-2.36	0
11	REACTOME_RESPONSE_OF_EIF2AK4_GCN2_TO_AMINO_ACID_DEFICIENCY	-2.33	0
12	REACTOME_TRANSLATION	-2.23	0
13	REACTOME_REGULATION_OF_EXPRESSION_OF_SLITS_AND_ROBOS	-2.16	0
14	REACTOME_DEPOSITION_OF_NEW_CENPA_CONTAINING_NUCLEOSOMES_AT_THE_CENTROMERE	-2.1	0
15	REACTOME_RRNA_MODIFICATION_IN_THE_NUCLEUS_AND_CYTOSOL	-2.08	0.001

16	REACTOME_MITOTIC_SPINDLE_CHECKPOINT	-2.05	0.002
17	WP_CHOLESTEROL_METABOLISM_INCLUDES_BOTH_BLOCH_AND_KANDUTSCHRUSSELL_PATHWAYS	-2.02	0.003
18	PID_MYC_ACTIV_PATHWAY	-2.02	0.003
19	PID_AURORA_B_PATHWAY	-2.02	0.003
20	REACTOME_CHOLESTEROL_BIOSYNTHESIS	-2.01	0.004
21	REACTOME_SIGNALING_BY_ROBO_RECEPTORS	-2	0.004
22	REACTOME_SNRNP_ASSEMBLY	-1.99	0.005
23	REACTOME_ACTIVATION_OF_GENE_EXPRESSION_BY_SREBF_SREBP	-1.98	0.006
24	PID_INTEGRIN1_PATHWAY	-1.97	0.007
25	REACTOME_KERATINIZATION	-1.96	0.007
26	REACTOME_RESOLUTION_OF_SISTER_CHROMATID_COHESION	-1.95	0.009
27	REACTOME_PROCESSING_OF_CAPPED_INTRON_CONTAINING_PRE_MRNA	-1.95	0.009
28	REACTOME_FORMATION_OF_THE_CORNIFIED_ENVELOPE	-1.94	0.009
29	KEGG_SPLICEOSOME	-1.92	0.012
30	REACTOME_POSTMITOTIC_NUCLEAR_PORE_COMPLEX_NPC_REFORMATION	-1.92	0.012
31	REACTOME_INTERCONVERSION_OF_NUCLEOTIDE_DI_AND_TRIPHOSPHATES	-1.9	0.015
32	REACTOME_REGULATION_OF_CHOLESTEROL_BIOSYNTHESIS_BY_SREBP_SREBF	-1.9	0.015
33	WP_CHOLESTEROL_BIOSYNTHESIS_PATHWAY	-1.89	0.016
34	REACTOME_INTERACTIONS_OF_REV_WITH_HOST_CELLULAR_PROTEINS	-1.89	0.016
35	WP_RETINOBLASTOMA_GENE_IN_CANCER	-1.89	0.016
36	REACTOME_TRNA_PROCESSING_IN_THE_NUCLEUS	-1.88	0.016
37	REACTOME_MRNA_SPLICING	-1.87	0.018
38	REACTOME_G0_AND_EARLY_G1	-1.87	0.018
39	REACTOME_NUCLEAR_PORE_COMPLEX_NPC_DISASSEMBLY	-1.86	0.021
40	REACTOME_NON_INTEGRIN_MEMBRANE_ECM_INTERACTIONS	-1.85	0.021
41	REACTOME_POSITIVE_EPIGENETIC_REGULATION_OF_RRNA_EXPRESSION	-1.84	0.023
42	WP_TGFB_SIGNALING_IN_THYROID_CELLS_FOR_EPITHELIALMESENCHYMAL_TRANSITION	-1.81	0.033
43	KEGG_TGF_BETA_SIGNALING_PATHWAY	-1.81	0.033
44	REACTOME_MITOTIC_PROMETAPHASE	-1.81	0.033
45	REACTOME_CELL_CYCLE_MITOTIC	-1.81	0.034
46	WP_MRNA_PROCESSING	-1.79	0.04
47	BIOCARTA_CELLCYCLE_PATHWAY	-1.78	0.043
48	REACTOME_CHROMOSOME_MAINTENANCE	-1.78	0.043
49	PID_E2F_PATHWAY	-1.78	0.043
50	WP_HYPOTHESIZED_PATHWAYS_IN_PATHOGENESIS_OF_CARDIOVASCULAR_DISEASE	-1.77	0.043
51	REACTOME_POLO_LIKE_KINASE_MEDIATED_EVENTS	-1.77	0.043
52	WP_METABOLIC_REPROGRAMMING_IN_COLON_CANCER	-1.77	0.043
53	REACTOME_INTERACTIONS_OF_VPR_WITH_HOST_CELLULAR_PROTEINS	-1.77	0.042
54	WP_GASTRIC_CANCER_NETWORK_1	-1.77	0.041
55	REACTOME_RNA_POLYMERASE_I_TRANSCRIPTION	-1.77	0.042
56	REACTOME_NS1_MEDIATED_EFFECTS_ON_HOST_PATHWAYS	-1.77	0.043
57	REACTOME_TRANSPORT_OF_MATURE_TRANSCRIPT_TO_CYTOPLASM	-1.76	0.043
58	WP_CELL_CYCLE	-1.76	0.042
59	WP_GENES_CONTROLLING_NEPHROGENESIS	-1.76	0.042
60	REACTOME_NUCLEAR_IMPORT_OF_REV_PROTEIN	-1.76	0.044
61	WP_CARDIAC_PROGENITOR_DIFFERENTIATION	-1.75	0.046
62	PID_INTEGRIN_A9B1_PATHWAY	-1.75	0.046
63	REACTOME_TRNA_PROCESSING	-1.75	0.046
64	NABA_ECM_GLYCOPROTEINS	-1.75	0.046
65	REACTOME_MRNA_DECAY_BY_3_TO_5_EXORIBONUCLEASE	-1.75	0.045
66	REACTOME_MITOTIC_METAPHASE_AND_ANAPHASE	-1.75	0.045
67	WP_REGULATION_OF_SISTER_CHROMATID_SEPARATION_AT_THE_METAPHASEANAPHASE_TRANSITION	-1.75	0.044
68	REACTOME_CELL_CYCLE_CHECKPOINTS	-1.74	0.046
69	KEGG_CELL_CYCLE	-1.74	0.046
70	WP_G1_TO_S_CELL_CYCLE_CONTROL	-1.74	0.046
71	REACTOME_EXPORT_OF_VIRAL_RIBONUCLEOPROTEINS_FROM_NUCLEUS	-1.74	0.047
72	WP_THE_EFFECT_OF_PROGERIN_ON_THE_INVOLVED_GENES_IN_HUTCHINSONGILFORD_PROGERIA_SYNDROME	-1.74	0.047

GSEA, gene set enrichment analysis; NES, normalized enrichment score; FDR, false discovery rate
